# Patient ancestry significantly contributes to molecular heterogeneity of systemic lupus erythematosus

**DOI:** 10.1172/jci.insight.140380

**Published:** 2020-08-06

**Authors:** Michelle D. Catalina, Prathyusha Bachali, Anthony E. Yeo, Nicholas S. Geraci, Michelle A. Petri, Amrie C. Grammer, Peter E. Lipsky

**Affiliations:** 1 AMPEL BioSolutions LLC & RILITE Research Institute, Charlottesville, Virginia, USA.; 2 EMD Serono Research & Development Institute, Billerica, Massachusetts, USA.; 3 Yeo Analytics LLC, Jersey City, New Jersey.; 4 Division of Rheumatology, School of Medicine, Johns Hopkins University, Baltimore, Maryland, USA.

**Keywords:** Autoimmunity, Rheumatology

## Abstract

Gene expression signatures can stratify patients with heterogeneous diseases, such as systemic lupus erythematosus (SLE), yet understanding the contributions of ancestral background to this heterogeneity is not well understood. We hypothesized that ancestry would significantly influence gene expression signatures and measured 34 gene modules in 1566 SLE patients of African ancestry (AA), European ancestry (EA), or Native American ancestry (NAA). Healthy subject ancestry-specific gene expression provided the transcriptomic background upon which the SLE patient signatures were built. Although standard therapy affected every gene signature and significantly increased myeloid cell signatures, logistic regression analysis determined that ancestral background significantly changed 23 of 34 gene signatures. Additionally, the strongest association to gene expression changes was found with autoantibodies, and this also had etiology in ancestry: the AA predisposition to have both RNP and dsDNA autoantibodies compared with EA predisposition to have only anti-dsDNA. A machine learning approach was used to determine a gene signature characteristic to distinguish AA SLE and was most influenced by genes characteristic of the perturbed B cell axis in AA SLE patients.

## Abstract 2

Transcriptional profiling of lupus patients and healthy controls reveals ancestry-related differences and transcriptional heterogeneity among lupus patients.

## Introduction

Systemic lupus erythematosus (SLE) is a complex, multigenic autoimmune disease affecting mostly women and characterized by autoantibodies to nucleic acids and nuclear proteins, leading to immune complex formation, complement deposition, and immune-mediated damage in multiple organ systems (1). The heterogeneity in ancestral prevalence, disease severity, organ involvement, and response to treatment has been described, but the explanation has not been fully delineated (2). Therefore, the development of transcriptomic signatures to determine the basis of ancestral differences in lupus disease expression is of great interest. Whereas the disease is most prevalent in Asians and people of African Ancestry (AA) (3–5), a disproportionate number of clinical trials have focused on the European Ancestry (EA) population (2, 6). Although not as extensively studied, native people of Native American ancestry (NAA) have also been shown to have earlier onset of disease and more organ involvement (7, 8). The Lupus in Minority populations: Nature vs. Nurture (LUMINA) study and others have demonstrated increased active disease, organ involvement, and autoantibody levels for AA compared with EA patients (9, 10), and other studies have shown increased mortality for AA patients (11, 12). At the cellular level, the AA population has been shown to have more activated B cells, CD27^–^IgD^–^ B cells, and B cell receptor signaling than the EA population (13). Several studies have demonstrated differences in responses of both innate immune cells as well as lymphocytes, suggesting that ancestral differences in immune cells may contribute to the different disease course and incidence between populations (14, 15). Ancestry-related differences in response to therapy have also been reported. AA SLE patients responded better to B cell depletion therapies than EA (16), but they displayed lesser responses to anti-BAFF treatment in a phase III clinical trial (17, 18). Higher serum levels of BAFF in AA SLE patients have led to the suggestion that higher doses of the biologic may be necessary in AA patients (19).

Heterogeneity in SLE gene expression signatures were first reported for the IFN-stimulated genes (20, 21), and an association of IFN signatures with autoantibodies has been reported (22–28). Kirou et al. (22) previously showed a significant association with anti-ribonucleoprotein (anti-RNP), anti-Smith (anti-Sm), anti–Sjögren’s syndrome–related antigen A (anti-SSA), and anti-dsDNA autoantibodies with an IFN gene signature (IGS) and that patients having multiple autoantibodies also were more likely to have an IGS. Further work to describe SLE patient gene expression differences has been carried out by creating modules of genes overrepresented in 158 pediatric SLE patients. Increased plasmablast, cell cycle, and erythroblast modules were detected in AA SLE patients, and increased myeloid signatures and inflammation were observed in EA and Hispanic SLE patients, suggesting that there may be an ancestral basis to explain some of the heterogeneity in SLE gene expression signatures (27). It is unknown whether adult SLE patients will have the same associations and whether other prominent gene expression signatures used to divide SLE patients into groups such as low-density granulocytes (LDG), granulocytes, T cells, B cells, and platelets will also have gene expression differences based on ancestry, but 1 group has separated adult lupus patients into 7 groups based on molecular signatures (29).

Whole blood (WB) gene expression analysis provides a relatively straightforward means of assessing a subject’s transcriptomic fingerprint. We sought to determine the contribution of ancestry, sex, standard of care (SoC) therapy, serology, and clinical manifestations to the WB gene expression profile of 1566 adult SLE subjects. This work provides strong evidence that much of the gene expression signature measured between SLE patients and healthy controls (HC) is related to patient ancestry resulting in alterations in the proportions of hematopoietic cells, cellular processes, and signaling pathways detected. Importantly, the ancestry-related variance in gene expression in healthy persons contributes to the differences observed in subjects with SLE.

## Results

### There is significantly different ancestral gene expression in SLE patients.

**Figure 1:**
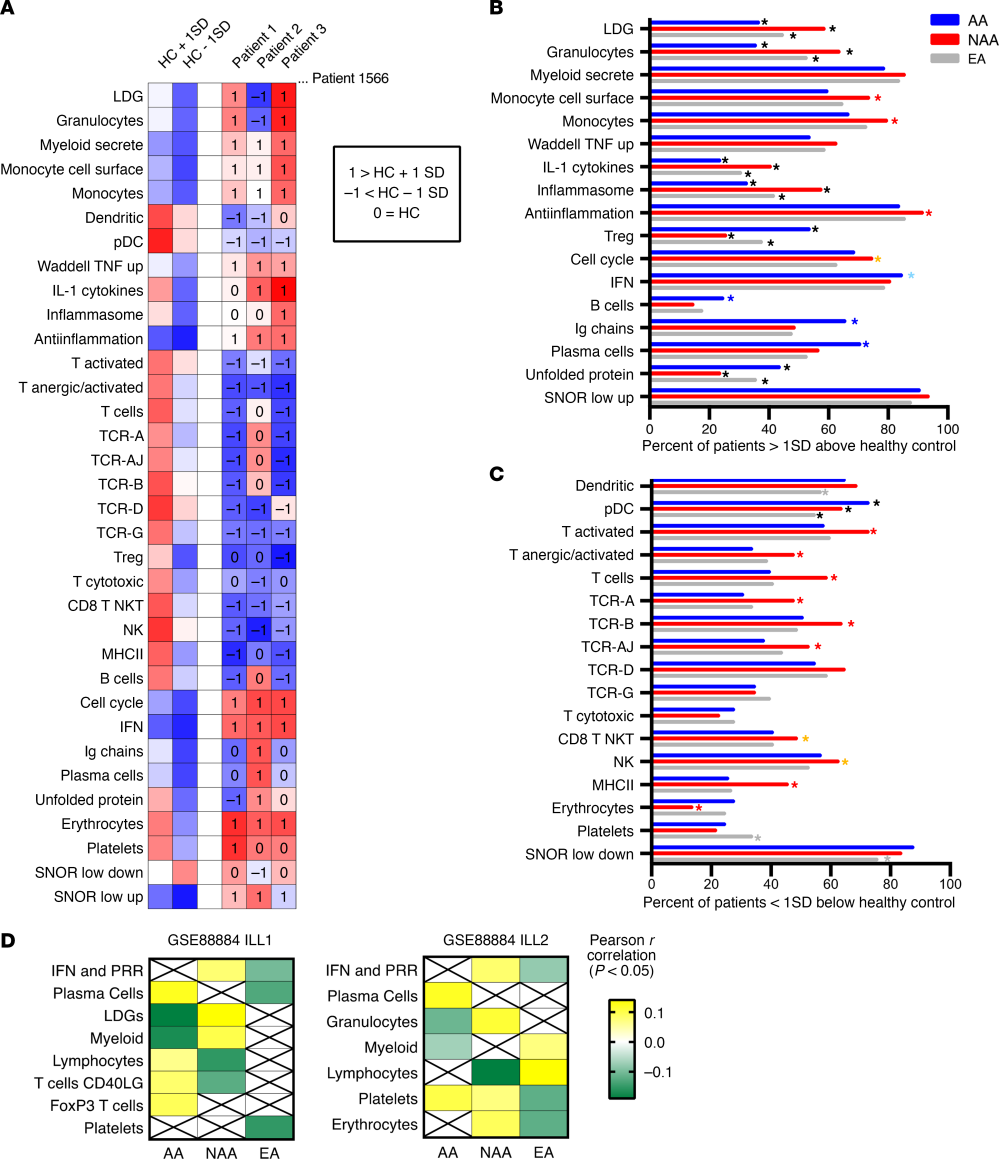
Individual SLE patients manifest varied patterns of signatures for 34 cell and process modules. (**A**) GSVA was carried out on 17 female HC to determine the mean and SD of control GSVA scores for 34 cell type and process modules. HC mean scores ± 1 SD were used to determine a normal range for GSVA scores. SLE female patient (GSE88884 ILL1 and ILL2 data set cohorts; *n* = 1566) GSVA scores were determined and compared with HC values to determine whether patients had increased (+1), decreased (–1), or normal (zero) values. GSVA enrichment gene symbols for each module are in Supplemental Table 5. (**B** and **C**) Percentage of patients within each ancestry (AA, *n* = 216; NAA, *n* = 232; EA, *n* = 1118) with > 1 (**B**) or < 1 (**C**) SD GSVA scores for each cell type and process module. Fisher’s exact *P* < 0.05 are indicated by different color asterisk: black asterisks for comparisons between all 3, red asterisks between NAA and AA/EA, orange asterisks between NAA and EA, light blue asterisks between AA and EA, and dark blue asterisks between AA and NAA/EA. Exact *P* values and percentages are listed in Supplemental Table 6. (**D**) WGCNA was carried out on data set GSE88884 ILL1 and ILL2 cohorts separately. Pearson correlation *r* values to ancestry were determined for each module and listed if *P* < 0.05.

In order to compare the ancestral contribution to gene expression, we made use of 2 large phase III clinical trial gene expression data sets (Illuminate 1 [ILL1] and ILL2; GSE88884) with a minimum disease severity requirements of the Systemic Lupus Erythematosus Disease Activity Index (SLEDAI) ≥ 6 and positive antinuclear autoantibody (ANA) that were well matched for average, median, and range of SLEDAI and percentage of patients with anti-dsDNA between AA, EA, and NAA SLE patients (Supplemental Table 1; supplemental material available online with this article; https://doi.org/10.1172/jci.insight.140380DS1). These ancestral groups were also well matched for SLE manifestations used to determine SLEDAI (Supplemental Table 2) (30–32). Bulk differential expression (DE) analysis of ILL1 determined there were thousands of differentially expressed genes (DEGs) between 798 SLE patients of AA, EA, and NAA, but no differentially expressed transcripts when each ancestry was randomized into 2 groups and compared with itself. These differences were reproduced in a second cohort of 768 patients (ILL2) and then confirmed in another unrelated data set (GSE45291) of 244 low–disease activity AA and EA SLE patients who were also matched for mean, median, and range of SLEDAI and ANA titer (Supplemental Tables 3 and 4; Supplemental Figure 1). We sought to determine how individual patient signatures contributed to these stable, reproducible group differences between ancestries. We employed gene set variation analysis (GSVA) with gene expression data from 1566 female AA, EA, or NAA SLE patients (GSE88884 data set cohorts ILL1 and ILL2) (31) to compare enrichment of 34 gene modules corresponding to lymphocytes, myeloid cells, and cellular processes (Figure 1A and Supplemental Table 5; ref. 33). We have previously used GSVA modules representative of cellular types and processes to determine enrichment in SLE patients and mice (33, 34). GSVA is advantageous compared with gene set enrichment analysis (GSEA) because it does not require a priori designation of 2 groups on the basis of phenotype and is helpful when disease samples are highly heterogeneous and there are low numbers of control samples (35). GSVA demonstrated that NAA had the highest percentage of patients with enrichment of LDG, granulocyte, IL-1, and inflammasome signatures followed by EA patients, and AA had the lowest. NAA also had significantly more patients with enrichment of monocyte cell surface and monocyte modules than AA patients, but notably, signatures for myeloid-secreted proteins, which included complement components *TNF* and *CXCL10*, were not different between the 3 ancestries. AA had significantly more patients with B cell, Ig, plasma cell, and Treg signatures compared with EA and NAA. NAA patients had significantly fewer patients with T cell–associated signatures compared with both EA and AA, whereas EA had significantly fewer patients with decreased DC and plasmacytoid DC (pDC) signatures compared with controls. The percentage of AA patients with enrichment of the IGS was higher than EA. AA and NAA had significantly fewer patients with decreased erythrocyte and platelet GSVA scores compared with EA (Figure 1, B and C, and Supplemental Table 6). GSVA scores for the 34 cell and process modules were also calculated for 14 AA, 93 EA, and 17 NAA male patients and male HC in SLE data set GSE88884. The pattern of enrichment was similar to that observed for the 1566 females in Figure 1B with increased plasma cells, Ig, and Treg signatures in AA SLE patients and increased LDG and myeloid signatures in and EA SLE patients, although statistical significance between the groups was noted only for the LDG, granulocyte, Treg, TCRA, TCRB, and platelet signatures (Supplemental Figure 2A and Supplemental Table 7).

Weighted gene coexpression network analysis (WGCNA) confirmed the association of ancestry with cellular signatures. WGCNA of female patients from the 2 cohorts of data set GSE88884 was carried out separately and demonstrated a significant positive correlation of AA ancestry to plasma cell, T cell, and Treg gene modules and a significant negative correlation to granulocyte and myeloid cell modules. NAA ancestry exhibited positive correlations to IGS, granulocyte, platelet, and erythrocyte modules and negative correlations to T cell and lymphocyte modules. EA ancestry was positively correlated to 1 myeloid cell module and negatively correlated to IGS, plasma cell, platelet, and erythrocyte modules (Figure 1D and Supplemental Table 8). This, an orthogonal approach using coexpression-defined gene clusters, confirmed the ancestral-related gene expression differences.

### Ancestry provides the gene expression backbone for SLE gene expression abnormalities.

**Figure 2:**
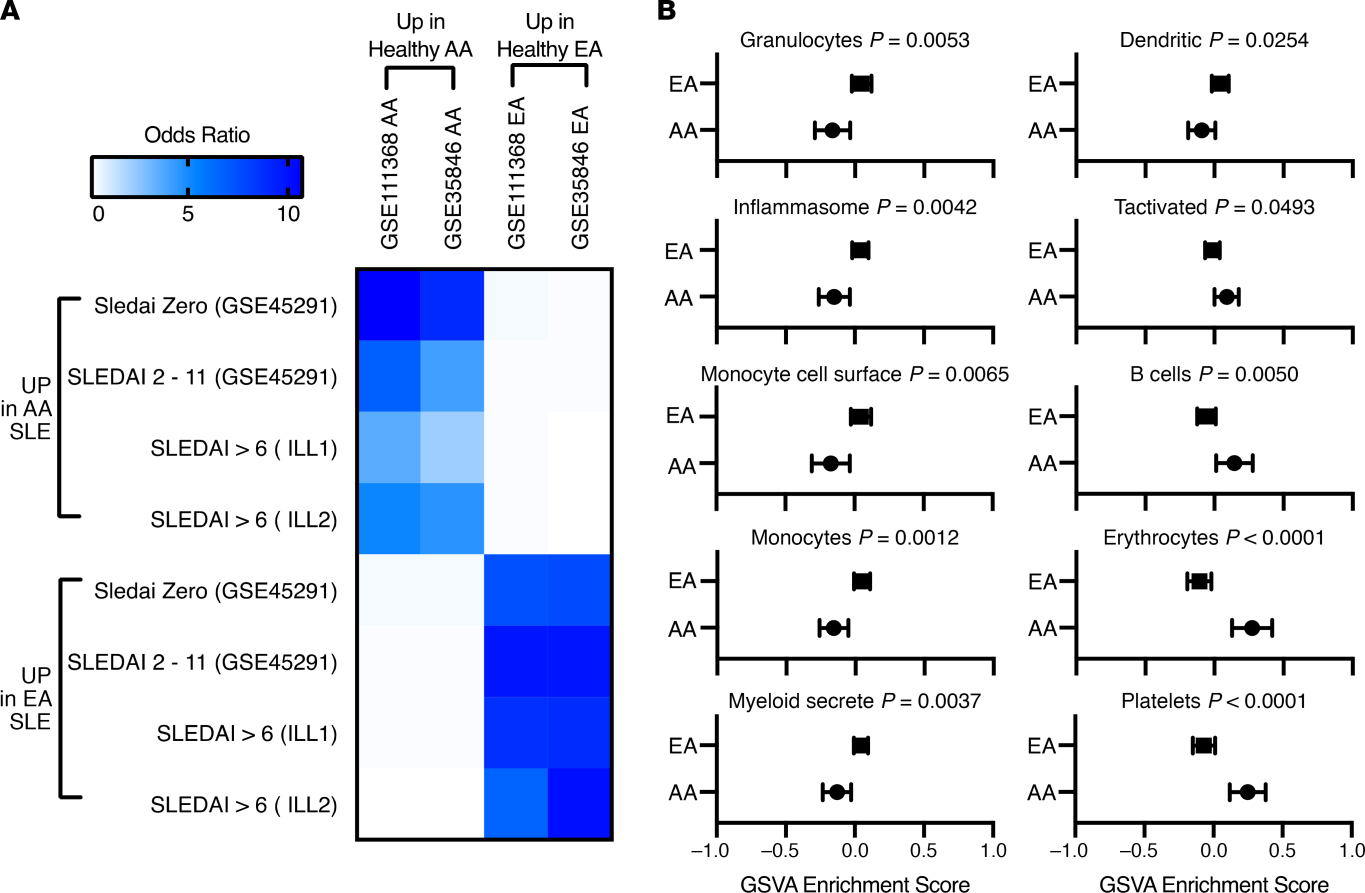
Gene expression differences in SLE patients are similar to ancestral gene expression differences in healthy controls. (**A**) Limma DE analysis was carried out between HC AA and EA for 2 separate data sets (Supplemental Table 9). Increased (Up in AA) and decreased (Up in EA) transcripts were compared with 4 SLE cohorts of AA DE to EA. Overlap *P* values were all below 1 × 10^–22^ for OR above 1. (**B**) GSVA for the 34 cell and process modules was carried out on healthy AA and EA subjects from 2 separate data sets. Welch’s *t* test was used to determine significant differences between ancestral GSVA scores; the mean and CI for the 10 GSVA scores significantly different (*P* < 0.05) between ancestries are shown.

Analyses of DEGs detected between different ancestries showed that AA populations had decreased expression of the Duffy blood group antigen *ACKR1*, the platelet, dendritic, and monocyte receptor *CD36*, and *G6PD* in comparison with NAA and EA populations (Supplemental Table 4); these genes have previously been described as risk alleles resulting in decreased expression in AA (36–38). We hypothesized that ancestral-related gene expression differences detected between SLE patients may be related to heritable differences in expressed genes in hematopoietic cells of healthy subjects. In order to address this question, DE analysis was carried out between AA and EA healthy subjects from 2 separate data sets (Supplemental Table 9) and compared with the DEGs that differed between AA to EA SLE patients. There was a highly significant overlap in transcripts differentially expressed between healthy AA and EA subjects and transcripts differentially expressed between AA and EA SLE patients (Figure 2A). GSVA was carried out on the healthy AA and EA subjects, and enrichment scores were compared for the 34 cell and process modules. Ten of the 34 signatures were significantly different between AA and EA healthy subjects. Healthy EA subjects had significantly increased granulocyte, inflammasome, monocyte cell surface, monocyte, inflammatory secreted, and DC GSVA enrichment scores compared with AA healthy subjects, and AA healthy subjects demonstrated significantly increased T cell–activated, B cell, erythrocyte, and platelet GSVA enrichment scores compared with healthy EA subjects. No differences in LDG, plasma cell, T cell, IGS, or the other signatures were determined (Figure 2B). Thus, in the absence of disease, significant and reproducible gene expression differences exist between AA and EA and appear to be contributing to the molecular heterogeneity in gene expression.

### Autoantibodies and complement levels, but not other clinical features of lupus, were associated with significant changes in gene expression profiles.

**Figure 3:**
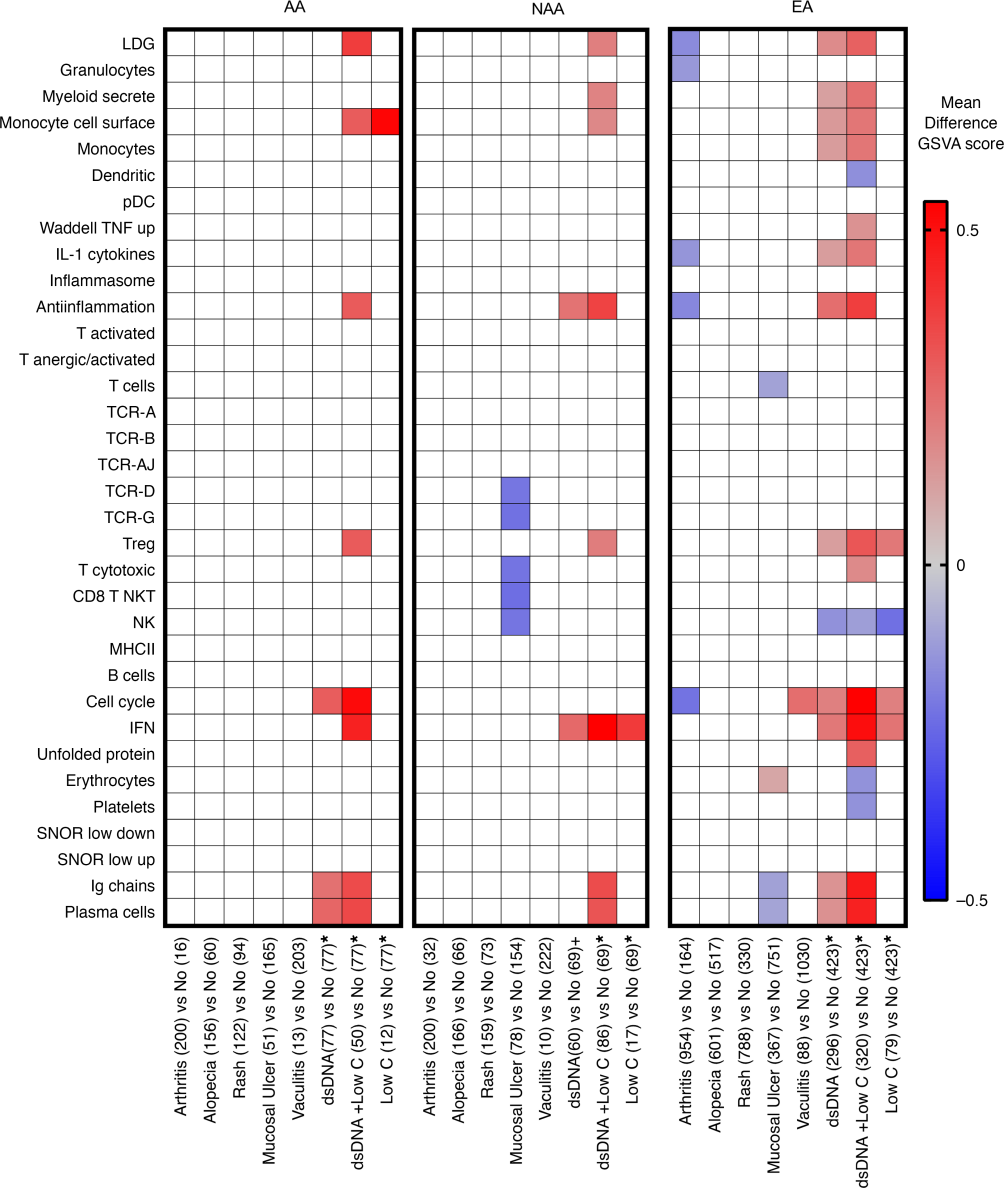
Autoantibodies and complement levels were associated with gene expression profiles. The mean difference in GSVA enrichment scores is shown for manifestations with significant (*P* < 0.05, Sidak multiple comparisons test) differences in enrichment scores as compared with all other manifestations. Asterisks indicate that, for dsDNA autoantibodies and low C, patients were compared with patients without either dsDNA autoantibodies (IU < 30) or low C (C3 > 0.8 g/L and C4 > 0.1 g/L). All patients in these analyses were positive for ANA. Number of patients with each SLEDAI component manifestation are shown in parentheses.

Variation in SLE disease manifestations has been reported as a potential cause for gene expression heterogeneity in SLE WB (27, 28, 39). However, the presence of arthritis, rash, alopecia, mucosal ulcers, or vasculitis had no consistent effect on cellular and process gene enrichment scores. Patients of all ancestries with both anti-dsDNA and low complement (low C) had significantly higher GSVA scores for antiinflammation, IGS, plasma cells, Igs, monocyte cell surface, and LDGs compared with patients without anti-dsDNA and low C (Figure 3).

**Figure 4:**
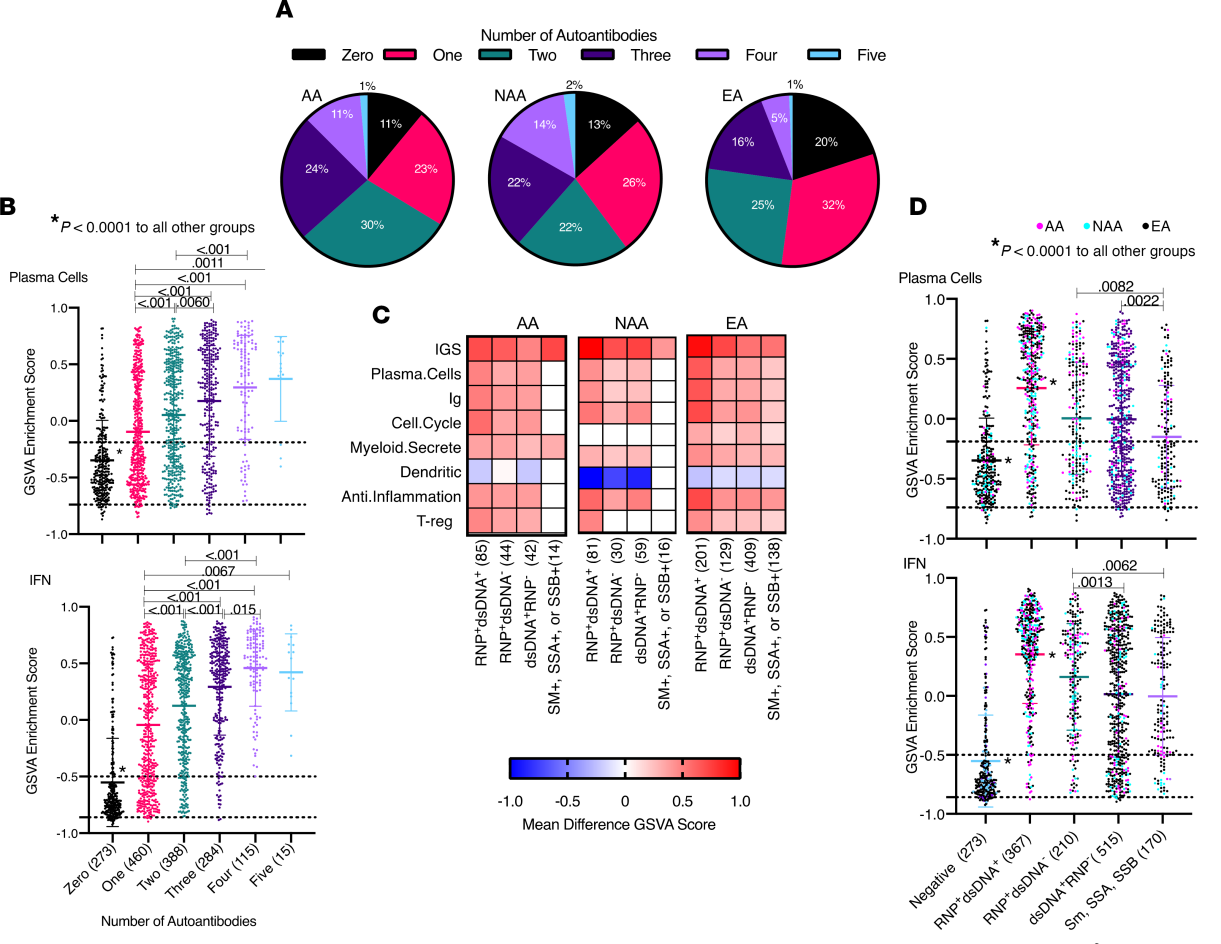
The higher number and different types of autoantibodies in AA SLE patients led to higher plasma cell, IGS, cell cycle, Treg, and myeloid-secreted signatures. (**A**) Percentage of patients with different numbers of 5 autoantibodies (RNP, Sm, SSA, SSB, and dsDNA) by ancestry. (**B**) Comparison of plasma cell and IGS GSVA scores by the number of autoantibodies. (**C**) GSVA enrichment scores for all 34 cell and process modules were compared, for each autoantibody group, with patients of the same ancestry with 0 of 5 autoantibodies. Tukey’s multiple comparisons test was used to determine significant differences; 8 cell and process module signatures had significant differences (*P* < 0.05) between autoantibody^+^ and autoantibody^–^ groups. (**D**) GSVA enrichment scores had significant differences between autoantibody groups. (**B** and **D**) Dots represent single patient scores, and data are presented as mean ± SD. Numbers of patients in each group are shown in parentheses. The black dotted lines represent the mean ± 1 SD of the HC for GSVA scores. Tukey’s multiple comparisons test was used to determine if significant differences existed between GSVA scores for plasma cells and IFN signatures for each group, and *P* < 0.05 are shown. RNP^+^ and/or dsDNA^+^ autoantibody groups could also have Sm, SSA, or SSB autoantibodies in any combination.

Notably, the significant increase in plasma cell signatures detected in AA patients could not be explained by AA SLE patients having an increased incidence of anti-dsDNA and low C; AA had the lowest number and percentage of patients with both anti-dsDNA and low C (23%), whereas 29% of EA and 37% of NAA had anti-dsDNA and low C. Anti-RNP and anti-Sm autoantibodies have been demonstrated to be increased in AA SLE patients (13, 40–42), and these autoantibodies could also be related to plasma cell, IFN, and other gene expression signatures. To understand how multiple autoantibodies change the transcriptome, we first determined the combinations of the 5 autoantibodies measured in this study for 1535 of the female SLE patients from ILL1 and ILL2: anti-dsDNA, anti-RNP, anti-Sm, anti-SSA, and anti-SSB. AA and NAA SLE patients had significantly higher frequencies of autoantibodies that are not dsDNA. Significantly fewer AA and NAA SLE patients were negative for all 5 autoantibodies compared with EA SLE patients. AA SLE patients had a significantly higher percentage with 3 or 4 autoantibodies and a significantly lower percentage of patients with only 1 autoantibody compared with EA, but there were no significant differences between AA and NAA. NAA SLE patients had significantly higher percentages of patients with 4 or 5 autoantibodies compared with EA (Figure 4A and Supplemental Table 10). Importantly, the presence of multiple autoantibodies was associated with significantly higher frequencies of the IGS and the plasma cell signature (Figure 4B).

For all 3 ancestries, patients positive for both anti-RNP and anti-dsDNA plus any of the other 3 autoantibodies had significantly increased enrichment scores for plasma cells, IGS, Ig, cell cycle, Treg, myeloid-secreted, and antiinflammation signatures compared with SLE patients negative for all 5 autoantibodies (Figure 4C). Additionally, patients positive for anti-RNP plus any of the other autoantibodies except anti-dsDNA had significantly increased IGS GSVA scores compared with patients positive for anti-dsDNA plus any other autoantibody and compared with patients with any combination of anti-Sm, SSA, and SSB (Figure 4D). These data explain the significantly increased plasma cell and IFN enrichment scores for AA SLE patients. AA SLE patients had significantly higher percentages of patients with anti-RNP autoantibodies (62%) compared with EA (30%) and NAA (51%), and significantly higher percentages of patients with anti-Sm (24%) compared with EA (12%) (Supplemental Table 11). AA also had significantly increased numbers of patients with both anti-RNP and anti-dsDNA compared with EA, and significantly increased numbers of patients with anti-RNP^+^ anti-dsDNA^–^ plus anti-Sm, SSA or SSB autoantibodies compared with EA and NAA. AA and NAA also exhibited more frequent SM, SSA, or SSB autoantibodies compared with EA (Supplemental Table 12). These data confirm, in a large cohort of AA, EA, and NAA SLE patients, ancestrally related disparities in autoantibody profiles, and they extend those findings to indicate that there is a significant association between autoantibody profiles and differences in gene expression between ancestries.

Autoantibody patterns in male SLE patients were similar to those determined in females, although statistical significance was not determined because of low patient numbers (Supplemental Table 13). Similar to female SLE patients, significantly increased IGS GSVA scores were determined for males with anti-RNP and anti-dsDNA plus any of the other 3 autoantibodies, and with RNP^+^dsDNA^–^ versus anti-dsDNA^+^ plus any of the other 3 autoantibodies, and all of these groups were significantly different from patients with none of these 5 autoantibodies (Supplemental Figure 2B).

### SoC therapy is associated with significant changes in gene expression profiles.

**Figure 5:**
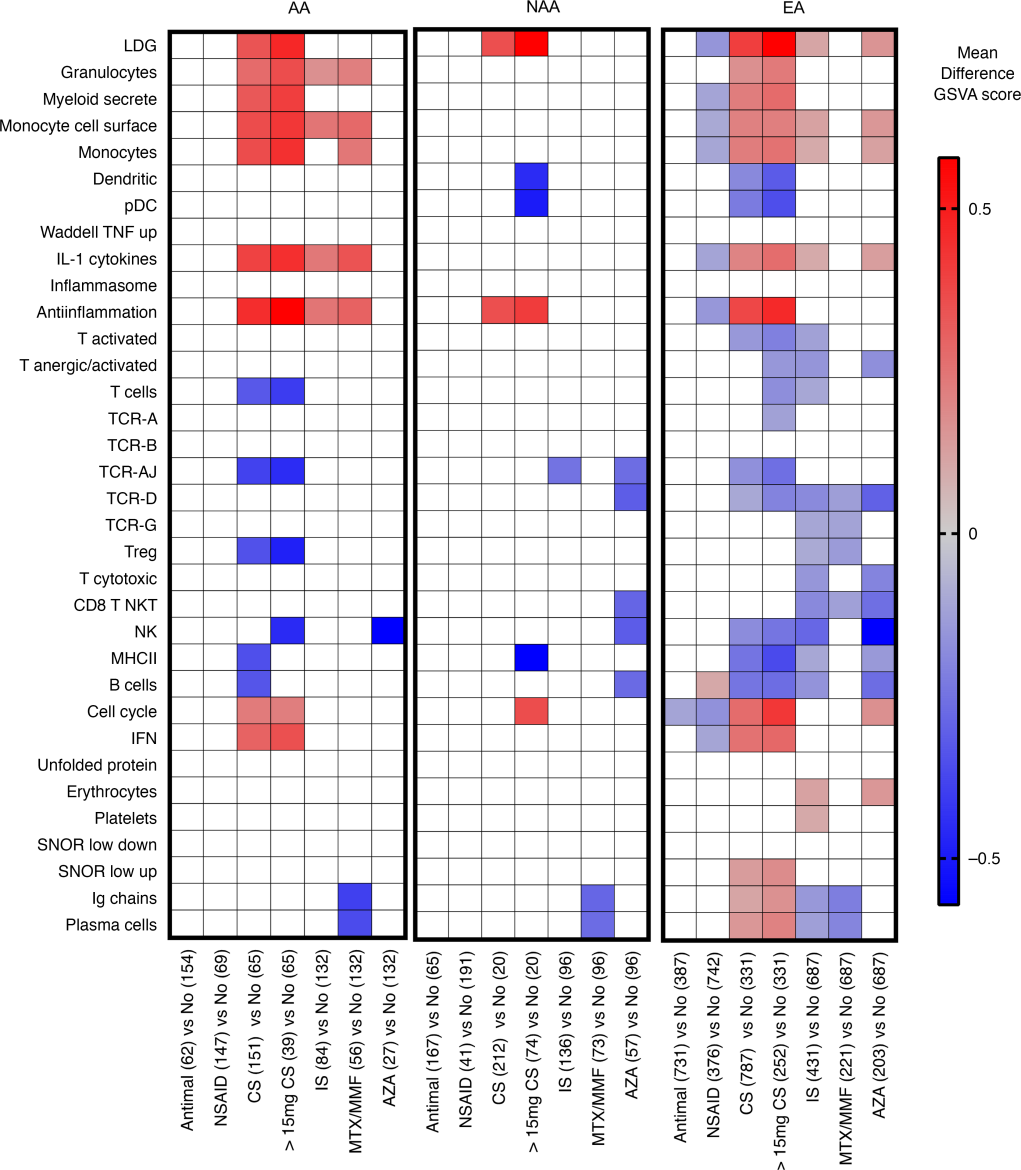
Association of corticosteroid (CS) use and immunosuppressive therapy with changes in gene expression profiles. Female SLE patients (1566 patients; GSE88884) were separated by ancestry and GSVA scores for each cell type or process module in patients receiving each therapy and were compared with GSVA scores for each cell type or process module in patients taking all other therapies. The patient numbers are in parentheses. Sidak multiple comparisons test was used to determine significant differences between therapies. The mean difference in GSVA score related to the treatment is shown for therapies with *P* < 0.05. Two EA patients were receiving cyclophosphamide and are included in the immunosuppressive (IS) calculation for EA.

SoC therapy has been demonstrated to significantly affect SLE gene expression signatures (27, 43), and significantly more NAA SLE patients were receiving corticosteroids (92%) and taking immunosuppressives (IS) (58%) compared with 70% and 39% of AA and 70% and 39% of EA patients, respectively (Fisher’s exact *P* < 0.0001). It was, therefore, important to consider therapy affects on gene expression and determine whether ancestry, SoC drugs, or both were contributing the differences in gene expression profiles. Corticosteroids significantly increased LDG and antiinflammation GSVA scores compared with patients of the same ancestry not taking the drugs. Additionally, both AA and EA receiving corticosteroids had significant enrichment of gene signatures for granulocytes, myeloid-secreted proteins, monocyte cell surface, monocytes, cell cycle, and the IGS. The effect of corticosteroids on myeloid signatures was further amplified at corticosteroid doses > 15 mg/day. When IS therapy was restricted to just mycophenolate mofetil (MMF) and methotrexate (MTX), there was a consistent decrease across all 3 ancestries in plasma cell and Ig GSVA scores. Because MTX and MMF were associated with low plasma cells scores, we compared the 5 autoantibody groups from Figure 4 for MTX and MMF usage and found no significant difference in usage between ancestral groups compared by autoantibody profile, demonstrating that ancestry-related autoantibody profiles, and not drugs, were related to differences in plasma cell signatures (Supplemental Table 14). Azathioprine (AZA) significantly decreased NK cell GSVA scores in all 3 ancestries and also significantly decreased T cytotoxic and B cell scores in NAA and EA ancestries. EA patients receiving NSAIDs compared with all other treatments had decreased LDG and antiinflammation signatures, whereas antimalarials had no significant effect on GSVA enrichment scores (Figure 5). Two separate cohorts of SLE patients with low disease activity from data set GSE45921 also had SoC drug information and were analyzed to confirm the findings. Corticosteroids increased LDG, monocyte, and antiinflammation GSVA scores; MTX and MMF decreased plasma cell GSVA scores; and AZA decreased NK and B cell GSVA scores (Supplemental Figure 3) in support of the data generated with the first data set composed of 1566 female SLE patients.

### Sex has a less important effect than ancestry on gene expression differences.

Because of the large number of EA females, we were able to balance the percentage of female and male patients on corticosteroids and IS in order to determine gene expression differences between male and female EA SLE patients (Supplemental Table 15). We also divided the females into 2 age groups, 25–49 years and > 50 years, because of the reported effects of estrogen on immune responses (44). There were very few differences between male and female SLE patients in gene expression (Supplemental Figure 4 and Supplemental Table 16), suggesting that ancestral differences are a more important factor in gene expression than sex differences.

### Logistic regression modeling demonstrated that ancestry is the major influence on SLE gene expression differences.

**Figure 6:**
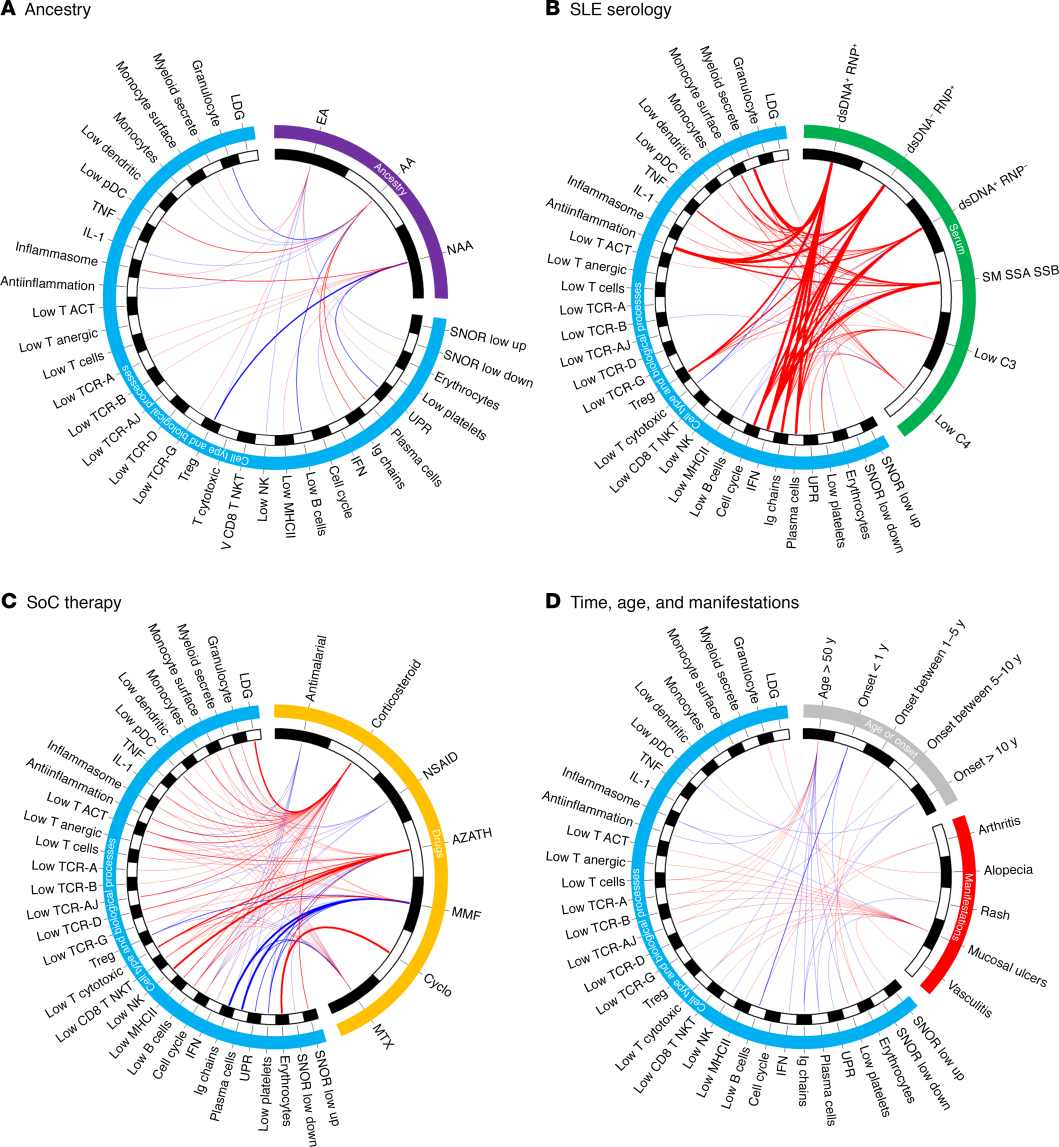
Stepwise logistic regression analysis determined the importance of ancestry, SoC drugs, and SLEDAI components to the gene expression profile. (**A–D**) CIRCOS visualization of odds ratios (OR) using stepwise logistic regression analysis for ancestry (**A**), serology (**B**), SoC drug (**C**), and time from onset of disease, age > 50, and SLE manifestation (**D**) to GSVA categories with *P* < 0.05 (*P* values, OR, and CI in Supplemental Tables 18–21). The thickness of the lines from the 26 variables to the GSVA categories represent the magnitude of the ORs. An interval graph was used to assign thickness of the lines where OR < 2, 1 pt; 2 ≥ OR < 3, 5pt; 3 ≥ OR < 10, 10pt; OR ≥ 10, 20pt. Red lines indicate OR above 1, and blue lines indicate OR below 1. OR between 0 and 1 are represented as 1/odds ratio to accurately reflect the magnitude of the negative relationship to the GSVA enrichment score.

To determine the relative importance of ancestry, SLE manifestations, serology, and SoC drugs on gene expression signatures, we performed stepwise logistic regression on data from 1535 female SLE patients with all 5 autoantibody measurements for each of the 34 cell type and process signatures using the variables of ancestry, SoC drugs, SLE serologic abnormalities, SLE manifestations, age, and time from onset of disease. Colinearity was excluded by carrying out Spearman’s correlations between all variables, and the ethnic term Hispanic was removed from modeling because of an r_s_ of 0.54 to NAA (Supplemental Table 17). Figure 6 shows CIRCOS visualizations of the OR for each variable significantly contributing to each GSVA score. Ancestry was associated with changes in 23 cell or process signatures. AA ancestry was positively associated with Treg, plasma cell, Ig, and low pDC signatures and negatively associated with granulocyte, monocyte, IL-1, antiinflammation, and low B cell signatures. NAA ancestry had the highest positive association to the inflammasome and a negative association to Treg signatures. NAA was also positively associated with erythrocyte, low T cell, and low MHC II and was negatively associated with the IGS and unfolded protein response signatures. EA was positively associated with high myeloid-secreted signatures, high inflammasome signatures, and low platelet signatures and associated negatively with low NK and Treg signatures (Figure 6A and Supplemental Table 18). SLE serologic profiles are interrelated to ancestral background and had the highest OR to significant changes in GSVA scores. Autoantibody groups RNP^+^dsDNA^+^, RNP^+^dsDNA^–^, RNP^–^dsDNA^+^, and any combination of Sm, SSA, and SSB, resulted in significant OR of 31.6, 25.6, 5.5, and 13.1, respectively, for the relationship to the IGS; OR of 7.9, 4.7, 4.0, and 2.3, respectively, for the relationship to the cell cycle signature; OR of 8.7, 3.9, 3.5, and 2.4, respectively, to the plasma cell signature; OR of 4.8, 3.0, 2.4, and 2.2, respectively, to the Treg signature; OR of 3.6, 2.4, 2.4, and 2.1, respectively, to the TNF signature; and OR of 9.0, 3.5, 2.6, and 3.4, respectively, to the myeloid-secreted signature. In total, autoantibodies and low C were related to changes in 23 cell and process signatures (Figure 6B and Supplemental Table 19). SoC drugs influenced every cell and process module GSVA score. Corticosteroids were significantly associated with increases in 14 cell and process signatures; the highest OR was 3.8 to the LDG signature. AZA was significantly associated with 9 signatures and had an OR of 4.8 to low NK cell signatures. Both MTX and MMF were associated with decreased lymphocyte signatures, especially plasma cells with OR of 0.394 and 0.211, respectively. (Figure 6C and Supplemental Table 20). Time, age, and clinical manifestations were associated with the fewest changes and the lowest ORs. Age > 50 was related to changes in 12 of the 34 cell type and process module, and the time from onset of disease was related to changes in 9 of the 34 cell type and process modules. Notably, a time of onset of < 1 year was negatively associated with low B cells, and age > 50 was negatively associated with the plasma cell signature. Clinical manifestations were related to changes in 17 cell type and process modules with mucosal ulcers related to changes in 13 modules; predominantly, those associated with low T cell signatures (Figure 6D and Supplemental Table 21).

The logistic regression model determined that multiple variables influenced each GSVA enrichment score, suggesting that simple linear regression between single variables and GSVA enrichment scores might not be useful. To test this, linear regression analysis was carried out between SLEDAI values, dsDNA titers, or C3 levels and the 34 cell and process gene modules in each ancestry (Supplemental Figure 6). Low or no relationship was found between SLEDAI and gene module GSVA scores. This is consistent with the finding that the individual clinical manifestations used to calculate SLEDAI (arthritis, rash, alopecia, mucosal ulcers, and vasculitis) had no or minimal relationship to changes in gene expression signatures, as determined by logistic regression. For single anti-dsDNA and C3, there was a relationship in all 3 ancestries to plasma cells, IFN, cell cycle, and LDG, but the predictive values of these values alone were not above 0.15.

### Machine learning identifies the perturbed B cell axis in AA SLE.

Ancestry was associated with significant changes in 23 of 34 gene expression modules, and additionally, the high OR for association of gene expression signatures with serologic components suggested that one aspect of ancestry was to bias the tendency to form multiple autoantibodies, including anti-RNPs. Comparison of GSVA enrichment scores of patients with and without specific therapies confirmed the logistic regression results, indicating that, while therapy had an important influence, ancestry was still a major contributor to gene expression profiles (Supplemental Figure 5). To confirm this conclusion, we carried out a machine learning approach to determine whether gene expression could predict AA in SLE and also to determine the major predictors of ancestry. Because NAA signatures in this study were biased by substantial drug therapy, they were not used, whereas AA and EA had similar drug therapy profiles (Supplemental Table 22)

**Figure 7:**
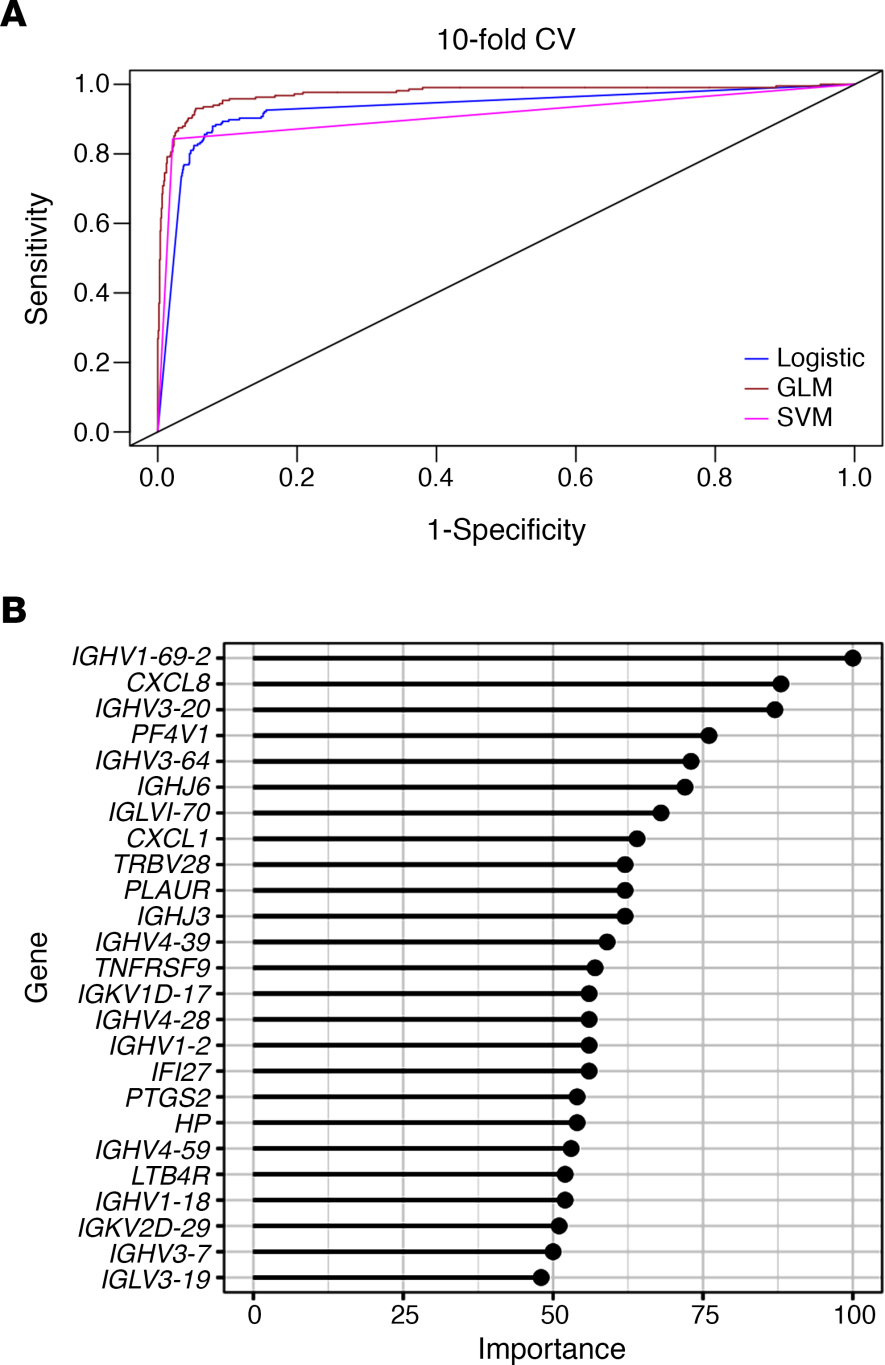
A machine learning approach predicted AA from EA SLE patients and demonstrated the perturbed B cell axis in AA SLE. (**A**) SLE patients were classified as AA using logistic regression, generalized linear models (GLM), and support vector machine (SVM) classifiers. ROC curve for logistic regression and the 2 different machine learning models in GSE88884 (ILL1 and ILL2 combined). (**B**) Top 25 gene predictors determined by SVM model.

Logistic regression and 2 different machine learning algorithms were used to distinguish AA SLE patients from EA SLE patients using the gene expression values for the list of 752 genes comprising the modules used for GSVA (Supplemental Table 5). Logistic regression analysis, an elastic generalized linear model (GLM), and Support Vector Machine (SVM) were deployed to predict the ancestry status of SLE samples and determine the top 25 predictors using the gene importance score. All 3 models showed good performance with minor differences in their highest and lowest accuracies in each data set. The SVM classifier was the strongest performer, with 97% and 96 % accuracy in ILL1 and ILL2, respectively. To ensure that models were not picking irrelevant information while learning the details in the training data, 10-fold cross validation was performed on each data set separately and also combining the 2 data sets. In both cases, the SVM outperformed the other classifiers with accuracy of 96% (Figure 7A and Table 1). The genes used to classify AA SLE compared with EA SLE reflect the perturbed B cells axis in AA SLE (Figure 7B). In a separate analysis, the same approach was used with the entire Illuminate data sets including the NAA subjects, and very similar results were obtained (Supplemental Figure 7).

## Discussion

This work demonstrated the significant impact of ancestry on gene expression patterns in SLE and by implication on the biologic pathways driving disease in patients of each ancestry. The increased plasma cell, IFN, Treg, and inflammatory cytokine signatures were most strongly related to the AA ancestral bias of having increased anti-RNP/SM autoantibodies and multiple autoantibodies. Additionally, AA was independently associated with plasma cells and Ig transcripts when modeled alongside autoantibodies, suggesting that AA SLE patients may have higher background levels of plasma cells. Furthermore, machine learning algorithms accurately identified AA SLE patients from their gene expression data and identified genes associated with B cells as important for distinguishing AA SLE. This is further evidence of the perturbed B cell lineage described in AA SLE patients (13, 19, 25, 40, 45), which relates to the increase in the healthy AA B cell axis and suggests a greater tendency for epitope spreading of the autoantibody repertoire.

Part of the ancestral variation in autoantibody specificities in SLE may be linked to HLA alleles, as demonstrated by the association between HLA-DRB1*03:01 and SSA/SSB autoantibodies in EA SLE patients (46). It is possible that there are AA predominant alleles that are strongly associated with the production of anti-RNP/Sm autoantibodies, as has been shown for the different AA and EA HLA alleles and their relationships to systemic sclerosis autoantibody profiles (47). These autoantibody profiles may be more strongly associated with RNP/Sm than SLE disease. One effort to address this question found a weak but positive association between the AA-associated SLE risk allele DRB1*15:03 and anti-RNP/SM autoantibodies (48). Further work including the inclusion of more AA-specific alleles in GWAS platforms, more AA SLE patients and controls, and standardized autoantibody testing may be needed to determine significant associations, as GWAS efforts in general have not included sufficient AA SLE patients (49).

Another potential explanation for increased autoantibodies to RNP/Sm in AA SLE patients may not have its etiology in the specificity of the antigen receptor/MHC interaction, but in the increased development of antibodies in general. AA HC have increased titers of antibodies compared with EA in response to vaccination to rubella (50), pertussis (51) and influenza (52). AA HC also have increased levels of IgG, IgA, and IgM compared with EA HC (53). Furthermore, multiple myeloma and its precursor disease, monoclonal gammopathy of undetermined significance, is increased in AA compared with EA (54), and these conditions develop from aberrant B cell differentiation into malignant plasma cells. Notably, many AA SLE patients in the current study had increased B cells compared with HC, HC AA had increased B cells compared with HC EA, and increased B cells in AA compared with EA has been previously reported (55). These data suggest some dysregulation in the B cell compartment, as there were no increased T cells associated with AA ancestry and T cells would be just as likely to be affected by the proportional decrease in myeloid cells detected in AA SLE patients. Because these ancestral differences in antibody formation, both healthy and aberrant, are not specific to SLE, it may be that the perturbed B cell axis in AA has its etiology in biogeographical immune-related allelic diversity (56) and could be directly related to antibody production and not disease phenotype. Finally, several ancestral-related genes divergent between AA and EA were differentially expressed between AA and EA HC, including *CXCL8*, *CXCL1*, *CXCL5*, *STAT1*, *CEPBP*, *ITGAM*, and *CD58* (15), providing evidence that ancestral-associated alleles may contribute to the gene expression profile. However, it should be noted that specific genes contributing to increased B cell activity and antibody generation have not yet been delineated.

AA SLE patients had decreased granulocyte, monocyte, pDC, and IL-1 signatures, and this is likely related to the ancestry-associated Duffy-null polymorphism (*ACKR1*) and benign neutropenia (36, 57), as healthy AA also had these signatures decreased compared with healthy EA. Importantly, a decrease in these signatures was not reflected in a decrease in signatures for myeloid inflammatory cytokines in AA SLE patients, and this suggests that, when HC of similar ancestry are used for comparison, signatures for granulocytes, monocytes, pDC, and IL-1 will not be different between AA SLE patients and controls. Increased transcripts associated with platelets were detected in both healthy and AA SLE in this study and have been previously reported (58), Reticulocytosis, which may account for the erythrocyte gene transcripts detected in our study, may be augmented in AA SLE patients because the ancestral *G6PD* deficiency may lead to induced hemolysis secondary to infection and leukocyte phagocytosis (59). Although there was no difference in AA and EA T cell GSVA enrichment scores, several genes reported to be hypomethylated in AA compared with EA CD4^+^ T cells were also overexpressed in AA compared with EA in both healthy and 3 of 4 SLE comparisons, including *IL32*, *CDKN1A*, *SLC2A1*, and *WIPI2*. We were not able to confirm the apoptosis-related genes reported to be hypomethylated in CD4^+^ T cells, as only *CDKN1A* and *TNFRSF10A* (2 SLE data sets) of the 10 apoptosis-related genes were differentially expressed between AA and EA controls or SLE patients (60). This may be because of the difficulty in detecting T cell–specific signatures in WB.

Whereas all of the increased LDG and monocyte signatures initially detected as increased in NAA turned out to be associated with corticosteroid usage, NAA was positively associated with increased inflammasome, erythrocytes, and the unfolded protein response and negatively associated with IFN, T cells, and MHC II. The NAA association with erythrocytes is of note, as an association of SLE and erythrocyte transcripts has been reported but could be related to ancestral background (27). EA was positively associated with low platelet, myeloid-secreted, inflammasome, NK cell, and SNOR low down signatures, a set of genes overexpressed to SLE patients in the ILL1 and ILL2 clinical trials that were initially grouped by the first principal component analysis (PCA) with HC but could distinguish this group from HC if compared without the other SLE patients.

Previous work has suggested a strong association between the IGS and autoantibodies (22) and the association of dsDNA autoantibodies with increased plasma cells (61) and Tregs (62) by flow cytometry. Our findings demonstrate that it is not the IGS that is ancestry dependent per se, as previously reported (26), but the presence of autoantibodies to RNP/Sm and the increased combination of autoantibodies that is associated with ancestry. Our findings demonstrate that AA patients are likely to have multiple autoantibodies in combination with anti-RNP autoantibodies, and in patients of any ancestry, more autoantibodies and anti-RNP autoantibodies were associated not only with an increased IGS and plasma cell signatures, but also Treg, cell cycle, and myeloid inflammation signatures. Previous work that did not find an increased association of anti-RNP with the IGS in AA SLE patients (40) is likely related to considering the autoantibodies one at a time instead of in combination. In addition to increasing our understanding of AA SLE, this work has strong implications for using anti-dsDNA to balance cohorts for clinical trial enrollment. The AA SLE patients entered into ILL1 and ILL2 looked similar by anti-dsDNA autoantibodies, but our work showed that this served to severely underestimate the contribution to the transcriptome and potentially to the disease severity of AA SLE patients. Multiple studies have demonstrated more aggressive disease and increased morbidity and mortality for AA SLE patients (9–12), and further work to understand whether the perturbed B cells axis and increased autoantibody diversity are directly related to the increased disease severity is required. Patients with IgG4-related disease who have more autoantibody diversity have more severe disease (63), suggesting a relationship between autoantibody diversity and increased disease activity. Because being single positive (for the 5 autoantibodies measured) was the most common finding for the 1100 EA SLE patients in the ILL1 and ILL2 phase III clinical trials, it suggests that anti-dsDNA is a good metric for EA autoantibodies but not AA or NAA autoantibodies.

Importantly, this work considered the combinations of the 5 autoantibodies to determine the effect of multiple autoantibodies on transcriptomic signatures. The significantly increased IGS in SLE patients of all ancestries with multiple autoantibodies, and the almost complete lack of the IGS in the 273 SLE patients without anti-RNP, -dsDNA, -Sm, -SSA, or -SSB provides support for the hypothesis that the IGS arises from downstream pattern recognition receptor signaling induced by endosomal TLRs binding to single- and double-stranded RNA and DNA containing immune complexes, as previously suggested (64). Autoantibody profiles may be heritable, and autoantibody associations for AA SLE patients have been demonstrated for alleles of *LRRC20*, *LPAR1*, *EFNA5*, and *VSIG2* to anti-SSB, anti-SSA/Sm, anti-RNP, and anti-RNP/Sm^–^, respectively (65). IFN appears to positively regulate TLR7 signaling and negatively regulate TLR9 signaling, suggesting that, in the case of chronic stimulation, RNA ligands for TLR7 will augment the IGS and DNA ligands will dampen the IGS (66, 67). Another potential contribution to the increased IFN signatures in patients with anti-RNP autoantibodies may be the extrusion of interferonogenic, oxidized mitochondrial DNA by neutrophils in response to anti-Sm/RNP autoantibodies (68, 69). Anti-RNP, -SSA, -Sm, and -SSB autoantibodies were also found more commonly in circulating immune complexes compared with anti-dsDNA autoantibodies, and immune complex endocytosis by Fc receptors may lead to efficient engagement of TLRs in endosomes and downstream IFN production (70).

This study highlights the importance of appropriate controls for gene expression studies, as the ancestral transcriptomic backbone may be emphasized depending on HC comparators. Two research groups have divided pediatric (27) and adult SLE patients (29) into 7 molecular groups based on their transcriptomic signatures, although the methodology for grouping and the resulting groups were not similar. These analyses suggest that different mechanisms and pathways may lead to similar clinical outcomes. Of note, there were no significant differences in SLEDAI values between the molecular groups (29). Our work suggests that some of these transcriptomic differences are likely related to ancestry or SoC medications and therefore may not be reflective of different molecular mechanisms of SLE. IFN, plasma cells, inflammation, cell cycle, and Treg signatures are highly related to autoantibodies and low C, distinct signs of SLE disease, but it will also be important to determine signatures related to other quantifiable metrics of SLE disease occurring in patients with low titers of autoantibodies. Our logistic regression analysis showed very low ORs for changes in signatures associated with manifestations other than serological measurements. This may suggest that disease processes manifesting in tissues may not change peripheral blood gene signatures to the extent detected for autoantibodies and low C, and it has implications for using molecular signatures to group patients for entry into clinical trials or for treatment.

The ancestral differences between males also appeared similar to the ancestral differences between females, suggesting the ancestral component to gene expression will be more important to consider than male/female differences. Major differences were reported in 1 lupus cohort between male and female SLE patients with respect to renal involvement and serological manifestations (71), but we detected few gene expression differences between males and females of EA ancestry when matched for SoC drugs.

SoC therapies affected every gene expression signature, and accounting for these effects is necessary to interpret blood transcriptomic signatures. SoC drug effects on the transcriptome were confirmed by reports in the literature for the elimination of circulating plasma cells by MTX and MMF (72, 73), elimination of NK cells by AZA (74), and an increase in circulating neutrophils by corticosteroids (75). In what may seem to contrast with previous reports (76, 77), we detected no association between the IGS and antimalarials; however, previous work looked at IFN protein and not the downstream signature, which may be retained in monocytes after the removal of IFN (33). NSAIDs have also been shown to block caspases and inflammation (78), and although the change in GSVA score was not greater than 0.2, there did appear to be a significant decrease in LDGs and the antiinflammation signature, at least in EA SLE patients. Corticosteroid usage had a significant effect on most myeloid-related gene signatures, and the most potent effect was on the LDG signature with an OR of 3.8. This relationship was also detected in SLE patients with SLEDAI values of zero, suggesting that it may not be related to increased disease activity, leading to corticosteroid use. This finding is in contrast to the proposed inflammatory role of LDGs in autoimmunity obtained from in vitro experiments (39, 68, 79). The relationship of corticosteroids to LDGs has strong implications against using this signature as a measure of disease severity or in interpreting LDGs as playing a role in worsening disease, as worsening disease might prompt an increase in corticosteroid doses.

It is important to emphasize that common signatures specific for SLE were detected and included genes associated with plasma cell, Ig, IGS, anti-inflammation, cell cycle, Treg, DC, TNF, and myeloid-secreted signatures. The balance of these SLE-related abnormalities was different in the various ancestral groups, and their prominence was clearly influenced by SoC medications. Despite this, when these influences were considered and mitigated, a set of molecular abnormalities consistent with SLE was discerned, as has been previously suggested (27, 33, 80). However, the interpretation of perturbations in gene expression profiles in subjects with SLE requires that all the individual influences, including ancestry, drug therapy, and serological manifestations, be considered, as each can have complex and often contradictory effects. Results from single cell technology will also be affected by ancestry and SoC therapy, and it will be important to separate out cell populations prominent in ancestries and induced or repressed by concomitant drugs, from cells actively participating in disease processes. Deconvolution of transcriptome data using ancestral, SoC drug, serologic impact, and SLE-specific signatures has the potential to stratify patients more effectively for therapy or entrance into clinical trials.

## Methods

### SLE patients.

Two large phase III clinical trial databases with baseline microarray analysis were analyzed (GSE88884; ref. 31). The ILL1 and ILL2 clinical trials had microarray expression data for 1566 female patients of self-described ancestry: AA (*n* = 216), EA (*n* = 1118), and NAA (mostly from South America [*n* = 232]; top 3 countries of origin Peru [*n* = 81], Ecuador [*n* = 30], and Guatemala [*n* = 27]) and 124 male patients of self-described ancestry: AA (*n* = 14), EA (*n* = 93), NAA (*n* = 17). Patients of other ancestries were removed to avoid low numbers of patients. Ancestral backgrounds were split evenly between the ILL1 and ILL2 data sets, allowing for a training and test set to determine gene expression differences. All patients had a positive ANA test and similar disease activity and percentage of patients with anti-dsDNA (30, 32) (Supplemental Table 1). The trials excluded patients with progressive lupus nephritis. Most patients recruited had a mixture of 6 SLE manifestations: arthritis (86.4%), anti-dsDNA (57.5%), low C (40.0%), alopecia (58.9%), rash (68.3%), and mucosal ulcers (31.7%) (Supplemental Table 2). The clinical trial database was made available by M.D. Linnik from Lilly. The SLE data set GSE45291 was also analyzed as 2 cohorts separated by SLEDAI. The first cohort was 73 AA and 71 EA SLE patients with the same range of SLEDAI scores (2–11), similar mean SLEDAI (AA 3.78 ± 2.46; EA 3.53 ± 2.08) and mode of SLEDAI (2). The second cohort were 25 AA and 75 EA, all with SLEDAI values of zero (Supplemental Table 3). M.A. Petri (Johns Hopkins) provided clinical and SoC drug information for data set GSE45291.

### Gene expression data sets.

Data were derived from publicly available data sets on Gene Expression Omnibus (GEO, https://www.ncbi.nlm.nih.gov/geo/). Raw data sources are as follows: GSE88884 female WB ILL1 (10 female HC, 798 SLE [540 EA, 101 AA, 157 NAA]; all with SLEDAI ≥ 6), GSE88884 female WB Illuminate 2 (ILL2; 7 female HC, 768 female SLE [578 EA, 115 AA, 75 NAA]; all with SLEDAI ≥ 6), GSE88884 male WB ILL1 SLE (5 male HC, 59 male SLE [6 AA, 42 EA, 11 NAA]), GSE88884 male WB ILL2 (4 male HC, 65 male SLE [8 AA, 51 EA, 6 NAA]); GSE45291 WB (9 female HC, female SLE [73 AA, 71 EA with SLEDAI 2–11]), GSE45291 WB (9 female HC, female SLE [25 AA, 75 EA]; all with SLEDAI equal to zero), GSE35846 WB from healthy females (55 EA, 22 AA), and GSE111368 WB from healthy females (10 AA, 57 EA).

### Quality control and normalization of raw data files.

Statistical analysis was conducted using R and relevant Bioconductor packages. For data sets GSE88884 (Affymetrix Human Transcriptome Array 2.0) and GSE45291 (Affymetrix HT HG-U133+ PM), nonnormalized arrays were inspected for visual artifacts or poor RNA hybridization using Affy QC plots. To increase the probability of identifying DEGs, analysis was conducted using normalized data sets prepared using both the native Affy chip definition files, followed by custom Brain Array Entrez chip definition files (CDFs) maintained by the University of Michigan Molecular and Behavioral Neuroscience Institute (Ann Arbor, Michigan, USA). The Affy CDFs include multiple probes per gene and almost twice as many probes as BA CDFs. Whereas Affy chip definition files can provide the greatest amount of variance information for Bayesian fitting, the Brain Array chip definition files are used to exclude probes with known nonspecific binding and those shown by quarterly BLASTs to no longer fall within the target gene. Illumina CDFs were used for the 2 Illumina HumanHT-12 V4.0 data sets (GSE35846 and GSE111368).

### DE.

Guanine Cytosine Robust Multi-Array Analysis (GCRMA) normalized expression values were variance corrected using local empirical Bayesian shrinkage before calculation of DE using the eBayes function in the open source BioConductor LIMMA package (81) (https://www.bioconductor.org/packages/release/bioc/html/limma.html). Resulting *P* values were adjusted for multiple hypothesis testing and filtered to retain DE probes with an FDR < 0.05 (82).

### Determination of female and male patients and controls.

Log_2_ expression values were used to determine sex of unknown HC and to compute sex module scores using the formula sex module = *XIST* log_2_ expression + *TSIX* log_2_ expression – (*UTY* log_2_ expression + *RPS4Y1* log_2_ expression + *USP9Y* log_2_ expression). Female controls scored above zero and male controls scored below zero. Five SLE patients with reported sex (3 male and 2 female) in GSE88884 ILL1 clinical trial database were found to have expression of genes consistent with the opposite sex (Supplemental Figure 8). For all analyses shown in this paper, patients were analyzed with their reported sex in the Illuminate clinical trial database. For Supplemental Figure 4 analysis of the gene expression differences between males and females, 3 males with inconsistent sex chromosome gene expression were removed and did not change the results.

### GSVA.

GSVA (35) (V1.25.0) is an open source software package available from R/Bioconductor (35) and was used as a nonparametric, unsupervised method for estimating the variation of predefined gene sets in samples of microarray expression data sets (www.bioconductor.org/packages/release/bioc/html/GSVA.html). The inputs for the GSVA algorithm were a gene expression matrix of log_2_ microarray expression values for predefined gene sets coexpressed in SLE data sets (Supplemental Table 5). GSVA scores were calculated nonparametrically using a Kolmogorov Smirnoff–like (KS-like) random walk statistic and a negative value for a particular sample and gene set, meaning that the gene set has a lower expression than the same gene set with a positive value. The enrichment scores were the largest positive and negative random walk deviations from zero, respectively, for a specific sample and gene set. GSVA calculates enrichment scores using the log_2_ expression values for a group of genes and normalizes these scores between –1 (no enrichment) and +1 (enriched).

Enrichment modules containing cell type and process-specific genes were created through an iterative process of identifying DE transcripts pertaining to a restricted profile of hematopoietic cells in 13 SLE microarray data sets and checked for expression in purified T cells, B cells, and monocytes to remove transcripts indicative of multiple cell types, as previously described (33). The TCRA, TCRB, TCRAJ, TCRD, TCRG, and Ig gene lists were taken from the Affymetrix HTA2.0 chip definition. SNOR down low were the 7 most decreased transcripts and SNOR up low were the 7 most increased transcripts compared with HC for 348 female patients from ILL1 and ILL2 SLE patients that did not separate from HC by principal component analysis (FDR < 0.005). The LDG signature was taken from purified LDGs DE to HC and SLE neutrophils (79) and consists mainly of neutrophil granule proteins from module B as described in Kegerreis et al. (43). The overlap in genes between some signatures was intentional and used to check that signatures were behaving cohesively in patients.

### WGCNA.

WGCNA (83) is an open source R package (https://horvath.genetics.ucla.edu/html/CoexpressionNetwork/Rpackages/WGCNA/).

Log_2_ normalized microarray expression values for the GSE88884 data set cohorts ILL1 and ILL2 were filtered using an IQR to remove saturated probes with low variability between samples and used as inputs to WGCNA (V1.51). Adjacency coexpression matrices for all probes in a given set were calculated by Pearson’s correlation using signed network type–specific formulae. Blockwise network construction was performed using soft threshold power values that were manually selected and specific to each data set in order to preserve maximal scale free topology of the networks. Resultant dendrograms of correlation networks were trimmed to isolate individual modular groups of probes, labeled using semirandom color assignments, based on a detection cut height of 1, with a merging cut height of 0.2, with the additional use of a partitioning around medoids function. Correlation to ancestry was performed using Pearson’s *r* against Module eigengenes (MEs), defining modules as either positively or negatively correlated with those traits as a whole.

### Gene overlap.

Gene overlap is an open source R bioconductor package (www.bioconductor.org/packages/release/bioc/html/GeneOverlap.html) used to test the significance of overlap between 2 sets of gene lists. It uses the Fisher’s exact test to compute both an OR and overlap *P* value. For comparison of data sets on different array platforms (Illuminate versus Affymetrix), FDR < 0.2 was used.

### Stepwise logistic regression modeling.

SAS 9.4 was used for stepwise logistic regression. GSVA enrichment scores greater or less than HC averages ± 1 SD were determined, and SLE patients were assigned a 1 or 0 based on having a signature greater or less (low) than HC. These scores were used as 34 dependent binary variables to be modeled individually as the outcome variable to 26 independent binary variables: ancestry (AA, EA, NAA), drugs (corticosteroids, anti-malarials, NSAIDs, AZA, MTX, MMF, Cyclophosphamide), SLE manifestations (rash, arthritis, mucosal ulcers, vasculitis, alopecia), autoantibodies and complement (anti-RNP^+^dsDNA^+^ plus any of SSA, SSB, or Sm; anti-RNP^+^dsDNA^–^ plus any of SSA, SSB, or Sm; anti-RNP^–^dsDNA^+^ plus any of SSA, SSB, or Sm; and SSA, SSB, or Sm, low C3, or low C4) and time (age > 50, time from onset of disease [≤1 year, >1 year ≤ 5 years, > 5 years ≤ 10 years, > 10 years]). Spearman’s correlation coefficients were determined between variables before stepwise logistic regression in order to determine whether groups were too similar to give independent information to the model (colinearity). The ethnic term Hispanic as a general category was removed since it had an r_s_ > 0.5 compared with NAA (Supplemental Table 17). The stepwise approach was used to produce the statistically significant model. The results of any model that violated the Hosmer Lemeshow test were discarded. The *P* values, OR, and CI are listed in Supplemental Tables 18–21.

### CIRCOS.

CIRCOS (V0.69.3) software was used to visualize the OR determined by stepwise logistic regression analysis. OR do not go below zero, and a change from an OR of 0.5 to 0.25 is the same relative change as that between 2.0 and 4.0. For representative visualization, OR between 0 and 1 were converted to the 1/X value where X is an OR between 0 and 1. An interval graph was used to assign thickness of the lines where OR < 2, 1pt; 2 ≥ OR < 3, 5pt; 3 ≥ OR < 10, 10pt; OR ≥ 10, 20pt.

### Machine learning analysis.

Logistic regression, an elastic GLM, and SVM were used to predict the ancestry status of SLE samples and determine the top 25 predictors using the gene importance score. R was used for implementation, as it is an open source statistical language with access to machine learning algorithms. Logistic regression, GLM, and SVM were implemented using glmnet, nnet, and e1071 R packages, respectively. The performance of the models was evaluated by 10-fold cross validation. This method avoids the problem of over-fitting by using all the observations for both training and validation by randomly assigning each patient to 1 of 10 groups. The model was fit using the first 9 folds for training and validated using the remaining 10th fold for testing. Similarly, each fold was validated. Performance metrics such as sensitivity and specificity were determined by averaging class probabilities from each fold. Receiver operating characteristic (ROC) curves and AUC were plotted and measured for each model using R.

### Data availability.

All microarray data sets in this publication are available on the NCBI’s database Gene Expression Omnibus (GEO) (https://www.ncbi.nlm.nih.gov/geo/). The data set accession numbers are GSE88884, GSE45291, GSE35846, and GSE111368.

### Code availability.

All bioinformatic software used in this publication is open source, freely available for R. Additionally, example code used in this paper for LIMMA, GSVA, and WGCNA are available at figshare, www.figshare.com File names are “AMPEL BioSolutions LIMMA Differential Expression Analysis Code”, “AMPEL BioSolutions Gene Set Variation Analysis Code”, and “AMPEL BioSolutions Weighted Correlation Network Analysis Code”.

### Statistics.

GraphPad PRISM 8 version 8.2.1 was used to perform mean, median, mode, SD, Tukey’s multiple comparisons test, Sidak multiple comparisons test, linear regression analysis, and unpaired, 2-tailed *t* test with Welch’s correction. *P* < 0.05 were considered significant. The Fisher’s exact test was performed in R.

### Study approval.

Publicly available data sets were used for this work. The IRB approvals are as follows.

Data set GSE88884: Clinical Trials NCT01205438 and NCT01196091 were conducted by Eli Lilly and Company. The protocol was approved by each IRB, subject to applicable laws and regulations and ethical principles consistent with the Declaration of Helsinki. IRB approval was obtained and written informed consent at each of the 186 clinical trial sites for NCT01205438 and the 192 study locations for NCT01196091 (31).

Data set GSE35846: All samples were obtained under written informed consent for participation in the Center for Health Discovery and Well Being study with the approval of the IRBs of Emory University (Atlanta, Georgia, USA) and the Georgia Tech (Atlanta, Georgia, USA) (84).

Data set GSE45291: The study protocol for SPARE (Study of biological Pathways, disease Activity and REsponse markers in patients with SLE) was approved by the Johns Hopkins University School of Medicine IRB. All patients provided written informed consent (28).

Data set GSE111368: The study was approved by the NHS National Research Ethics Service, Outer West London REC (09/H0709/52, 09/MRE00/67). Patients or their legally authorized representatives provided informed consent. Additional adult HC were recruited as part of a separate study and consented to their samples being used in additional studies (Central London 3 Research Ethics Committee, 09/H0716/41). Informed consent was obtained from all participants, and all relevant ethical regulations were followed (85).

## Author contributions

MDC performed data analysis, generated figures, and wrote the manuscript. PB and NSG performed data analysis and generated figures. ACG and MAP supervised data analysis. AEY carried out statistical analysis. MAP provided clinical data for GSE45291 and reviewed the manuscript. PEL supervised data analysis, directed the study, and wrote the manuscript.

## Supplementary Material

Supplemental data

Supplemental Tables 1-22

## Figures and Tables

**Table 1:**
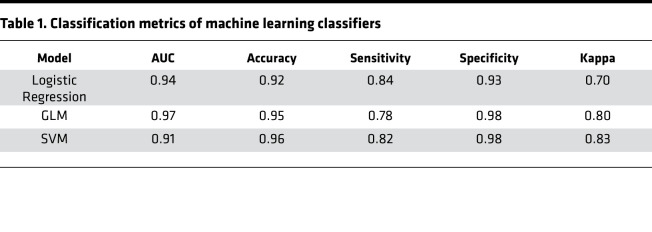
Classification metrics of machine learning classifiers

## Version 1 08/06/2020

Electronic publication
