# Supplementary material for: Patient ancestry significantly contributes to molecular heterogeneity of systemic lupus erythematosus
Source: JCI Insight. 2020 Aug 6;5(15):e140380. doi: 10.1172/jci.insight.140380 (PMC7455079; doi:10.1172/jci.insight.140380)
Supplement: Supplemental data [file jciinsight-5-140380-s226.pdf]

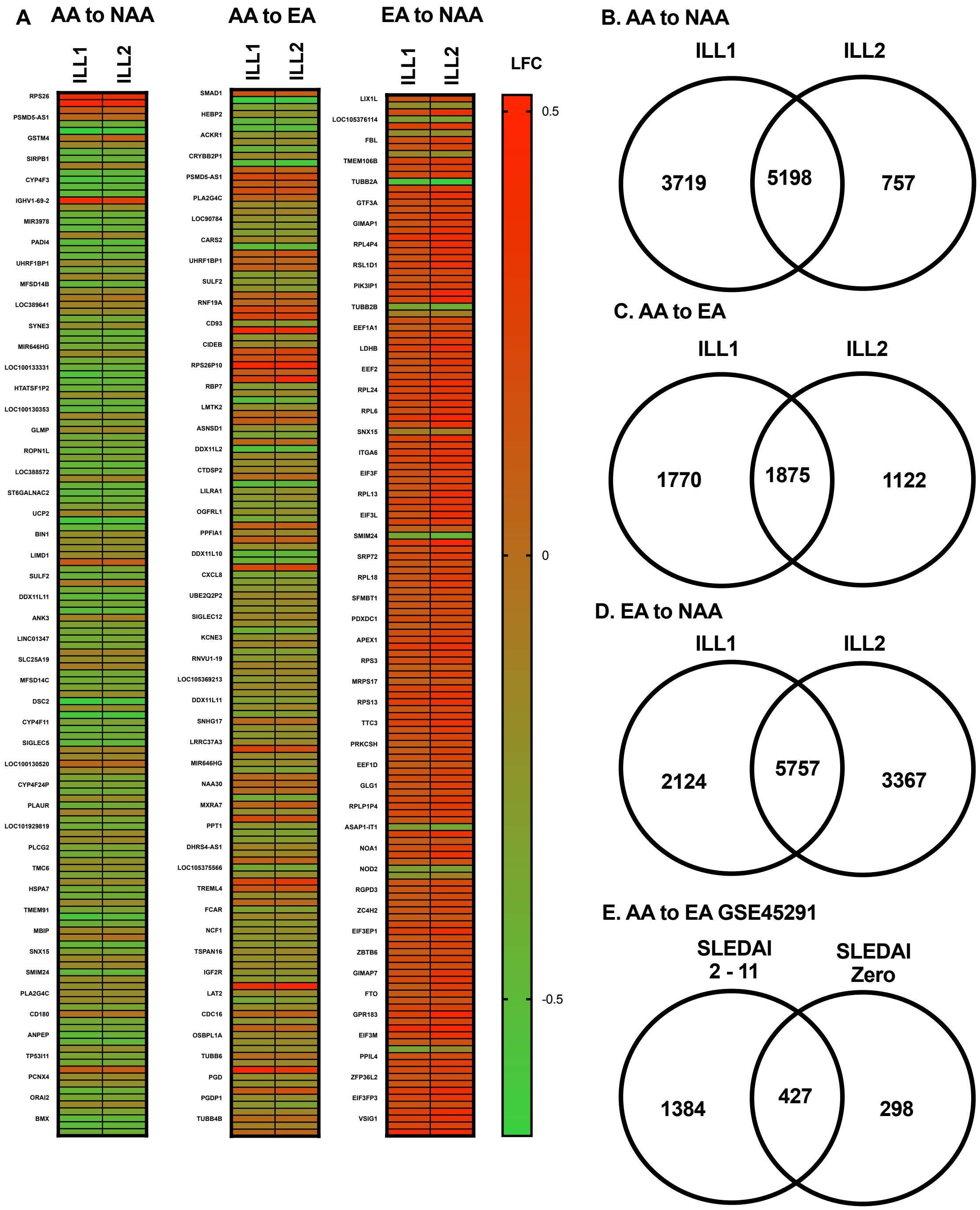

Supplemental Figure 1. SLE patients display ancestral gene expression differences.
(A) Limma differential expression (DE) (false discovery rate (FDR) < .05) between SLE patients of self described ancestry was carried out on two cohorts from dataset GSE88884: ILL1 (n = 101 AA, 540 EA, 157 NAA) and ILL2 (115 AA, 577 EA, 75 NAA). The top 150 genes by lowest FDR are shown for each comparison. **(B-D)** Venn diagrams depict the number of transcripts in common and unique between ILL1 and ILL2 for each ancestral comparison. Both increased and decreased transcripts were included. As a control, each ancestral background was randomized into two separate groups five separate times and no differentially expressed genes within the same ancestral background were found, even at a less stringent FDR < 0.2. **(E)** Limma DE analysis of SLE dataset GSE45291 for. 73 AA and 71 EA SLE patients with the same range of SLEDAI scores (2 – 11), similar mean SLEDAI (AA 3.78 +/- 2.46; EA 3.53 +/- 2.08) and mode of SLEDAI and DE analysis of 25 AA and 75 EA SLE patients with inactive disease (SLEDAI zero) were carried out. Venn diagram depicts the number of transcripts in common and unique between the AA to EA analysis in patients with SLEDAI 2 – 11 compared to patients with SLEDAI 0. The complete list of DE genes are in **Supplemental Table 4**.

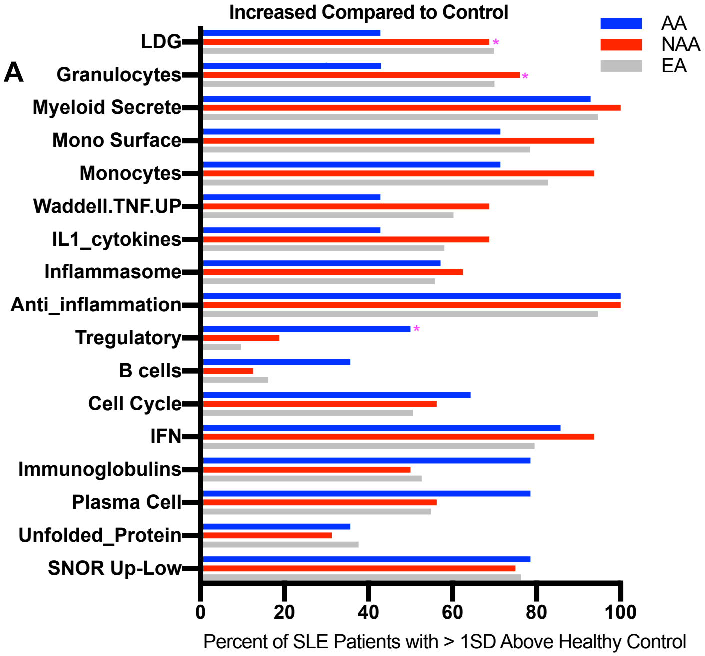

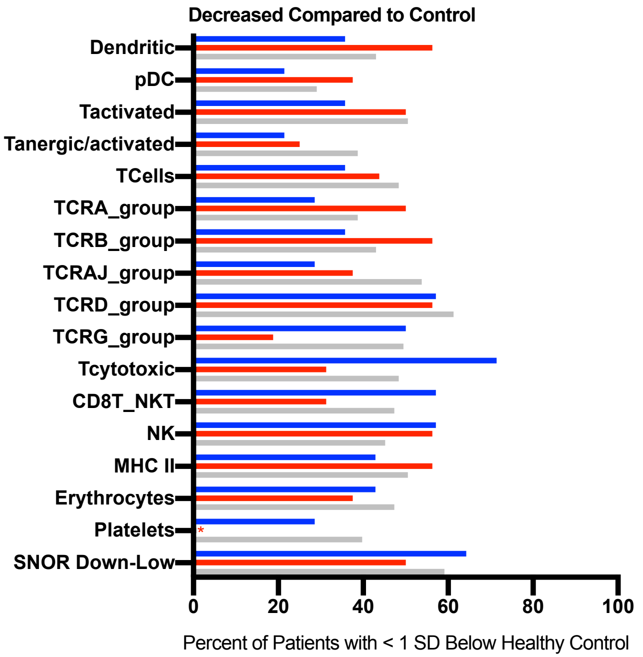

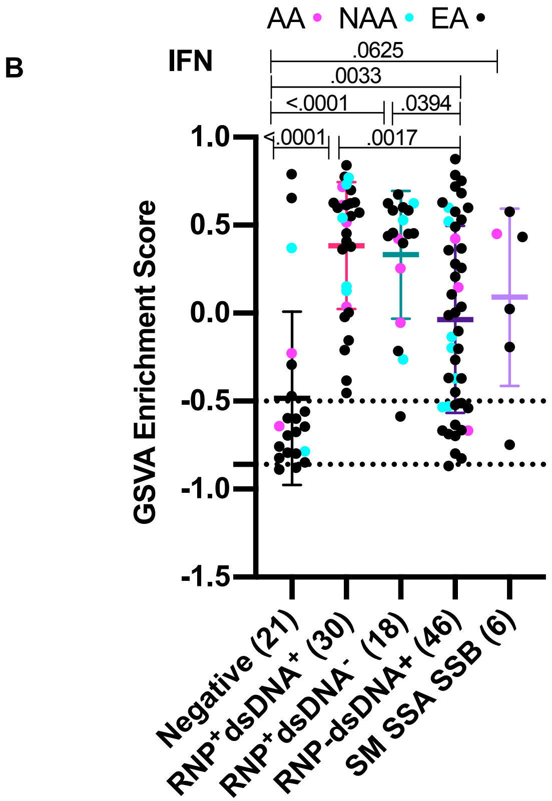

Supplemental Figure 2. Ancestral differences detected between females are similar in males. (A). Percentage of male patients within each ancestry (AA n = 14, NAA n = 17, EA n = 93) with > (top) or < than (bottom) 1 SD GSVA enrichment scores for each cell type and process module. Fisher's exact p values < .05 are indicated by different color *: pink * between AA and NAA, Dark blue * between AA and EA/NAA, red between NAA and AA/EA. **(B)** Comparison of male SLE patient IFN signature GSVA enrichment scores between five autoantibody groups (13 AA,16 NAA, 92 EA). n values are given in parentheses. Tukey's multiple comparisons test was used to compare the group GSVA scores and significant p values (p < .05) are shown on the graph. The RNP+dsDNA+, RNP+dsDNA- and RNP-dsDNA + groups could also be positive for any one of the three other autoantibodies measured, SM, SSA or SSB. The black dotted lines represent the mean plus or minus 1 SD of the healthy controls for GSVA scores.

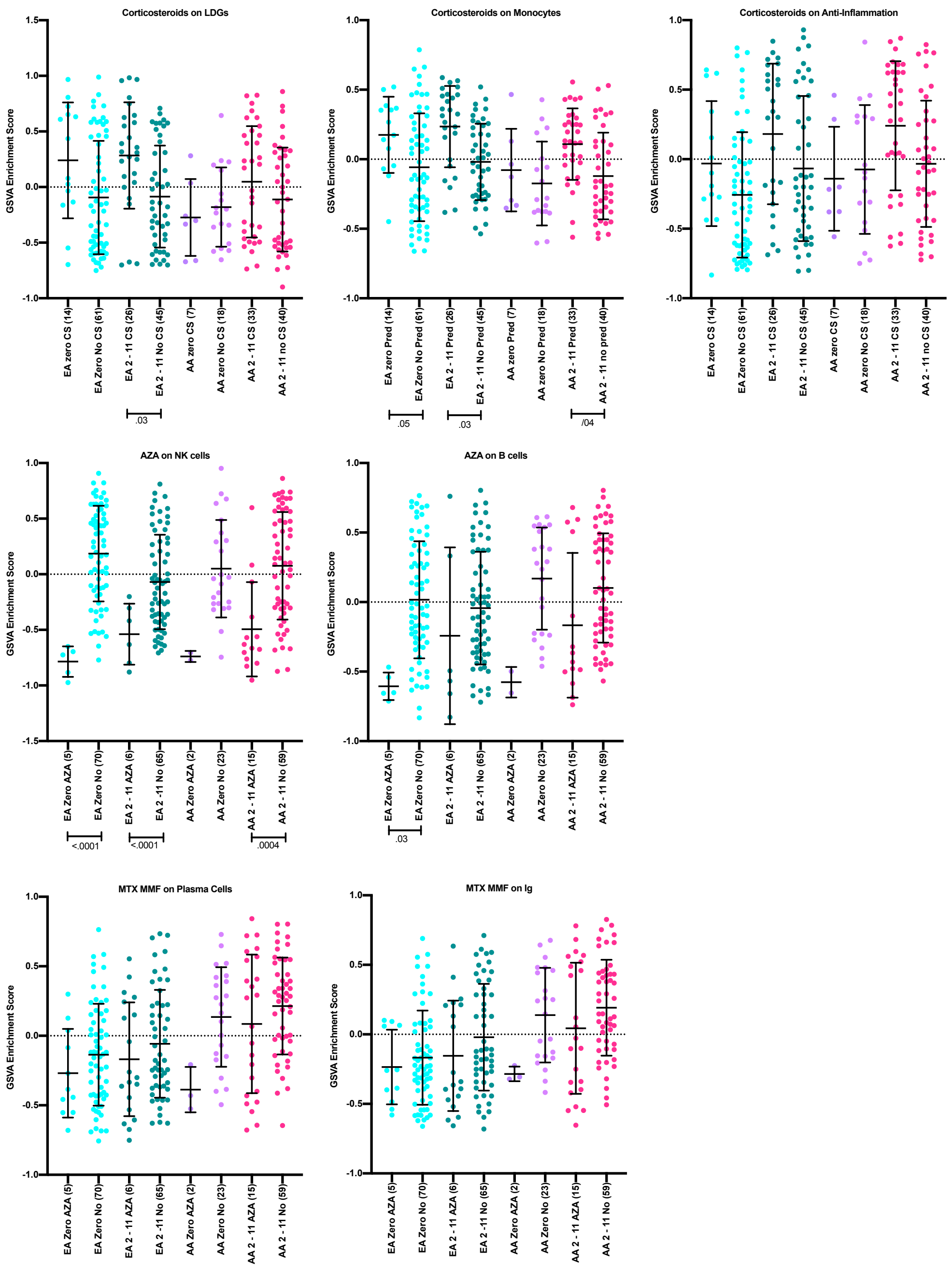

Supplemental Figure 3. Changes in GSVA scores associated with SoC drugs in dataset GSE45291. GSVA enrichment scores for AA and EA SLE patients with SLEDAI zero or SLEDAI 2 – 11 were compared using Tukey's multiple comparison's test. p values less than .05 are shown. Drug information was provided Michelle Petri. Number of patients for each comparison are in parentheses. CS (corticosteroids)

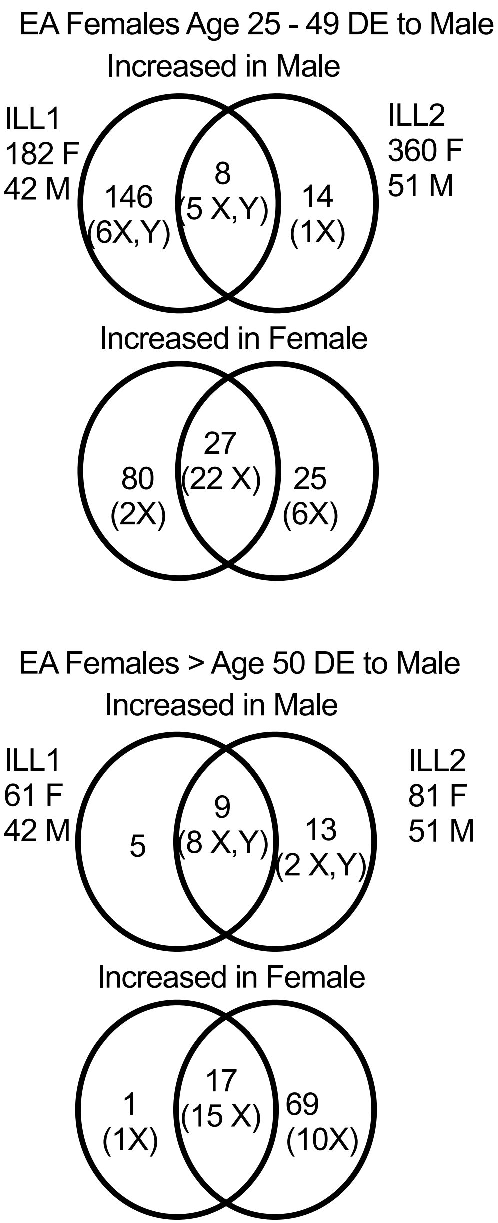

Supplemental Figure 4. Male and female SLE patients of the same ancestry with similar therapy profiles have few differences in gene expression. Female SLE patients were divided into two age groups, 25 – 49 years and > 50 years, to determine if estrogen effects on the immune response resulted in more DE transcripts compared to males. EA females age 25 – 49 or age > 50 years from the ILL1 and ILL2 datasets were used to make SoC drug matched groups for comparison to ILL1 and ILL2 EA males (**Supplemental Table 15**). The number of DE transcripts total and the number of transcripts on the X or Y chromosome are shown and the differentially expressed gene symbols and adjusted p values are listed in **Supplemental Table 16**.

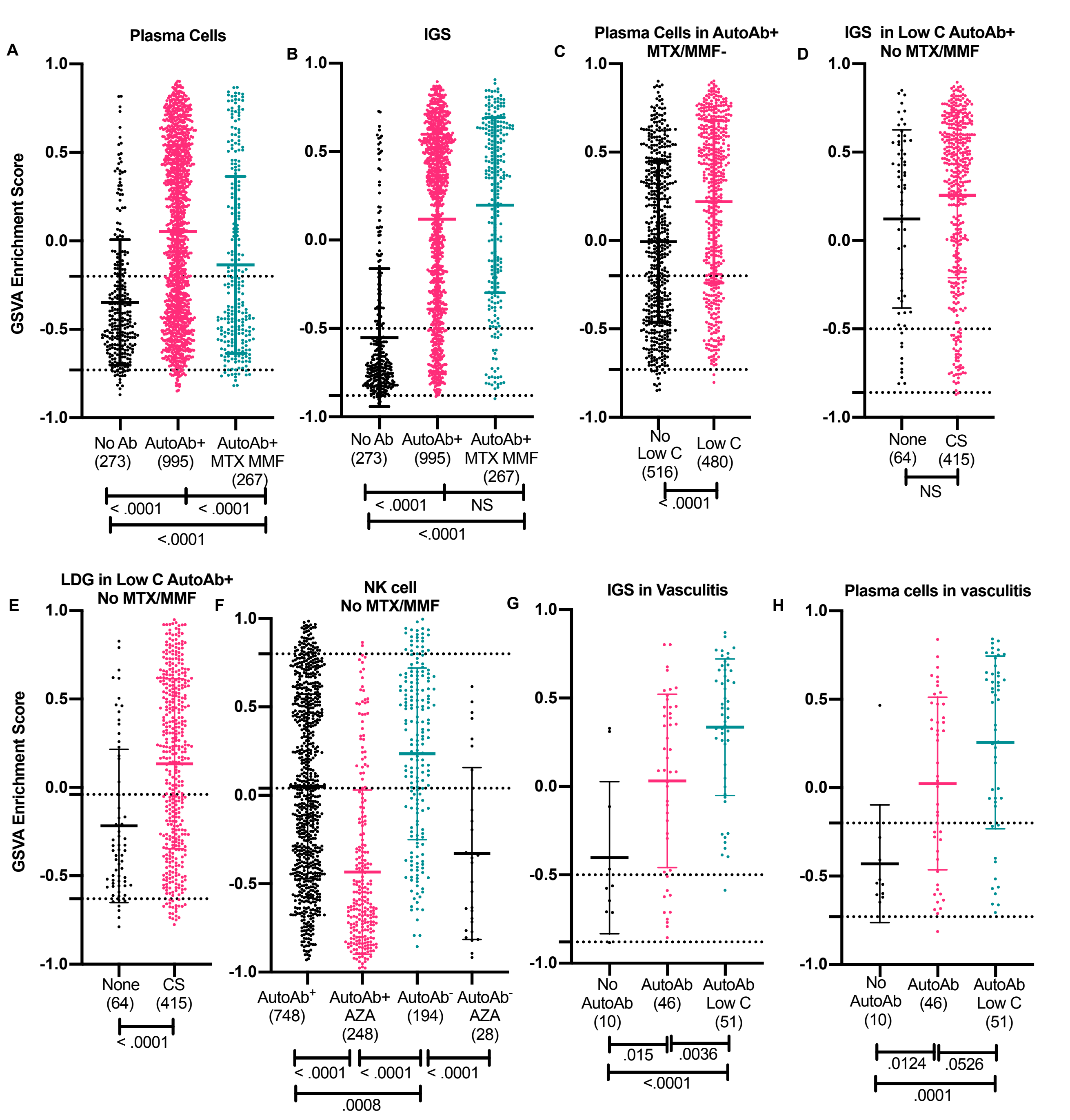

Supplemental Figure 5. Changes in GSVA scores associated with SoC drugs, autoantibodies and complement (**A – H**) Patients of all ancestries were used for these analyses. Tukey's multiple comparisons test was used to compare mean GSVA scores. p values for comparisons are shown below each graph. NS means not significantly different. n values in each category are in parentheses. Dots represent single patient scores and the error bars are mean and standard deviation. The black dotted lines represent the mean plus or minus 1 SD of the healthy controls. (**A,B).** Female patients in GSE88884 with five autoantibody (RNP, dsDNA, SM, SSA, SSB) measurements. Plasma Cell **(A)** and IGS **(B)** GSVA scores for patients with 0/5 autoantibodies (n = 273) were compared to patients with at least 1/5 autoantibodies (n = 922) and patients with at least 1/5 autoantibodies with concomitant MTX or MMF (n = 263). (**C)** The 922 patients positive for at least 1/5 autoantibodies were divided by the presence of low C (C3 ≤.8 g per L and/or C4 ≤ 0.1 g per L) and compared using the Welch's t-test. (**D,E)** The 487 SLE patients positive for at least one autoantibody and low C were divided by whether they were taking corticosteroids (CS) and IGS (**D**) and LDG (**E**) signatures were determined and compared using the Welch's t-test. **(F)** NK cell GSVA scores in patients with and without 1/5 autoantibodies and taking AZA. (**G,H)** All patients with vasculitis were divided by patients positive or negative for 1/5 autoantibodies and IFN and plasma cell GSVA enrichment scores were compared.

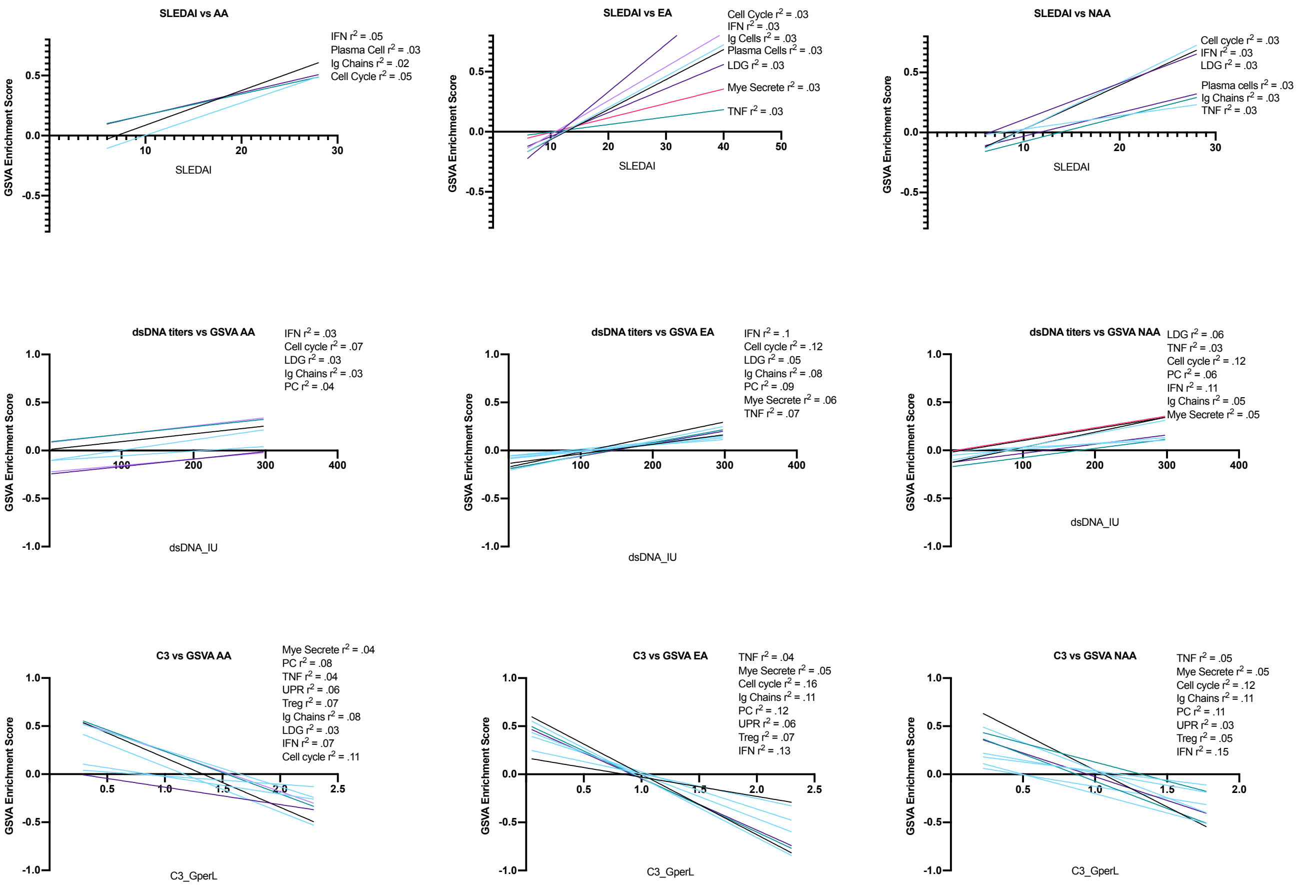

Supplemental Figure 6. Linear regression analysis between SLEDAI values, dsDNA titers (IU), and C3 gram/Liter (GperL) values and 34 cell and process module GSVA enrichment scores for female AA SLE patients (n = 208), EA SLE patients (n = 1100) and NAA SLE patients (n = 227). Only r^2 values for GSVA enrichment categories with $p < .05$ are shown.

A

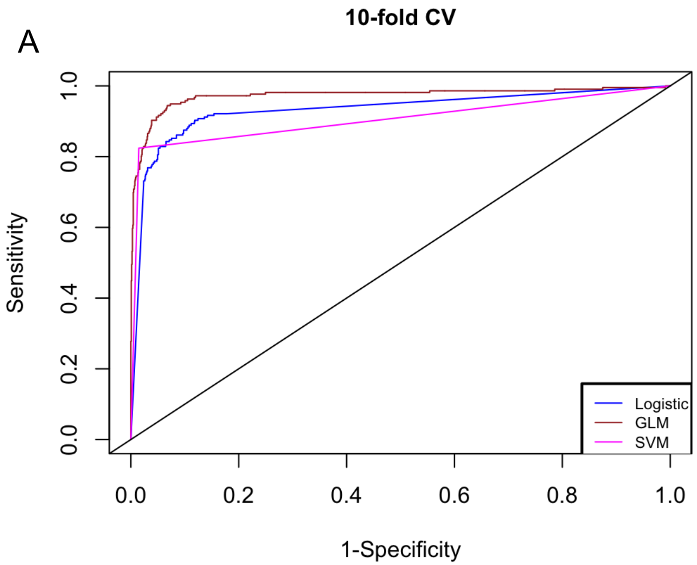

C

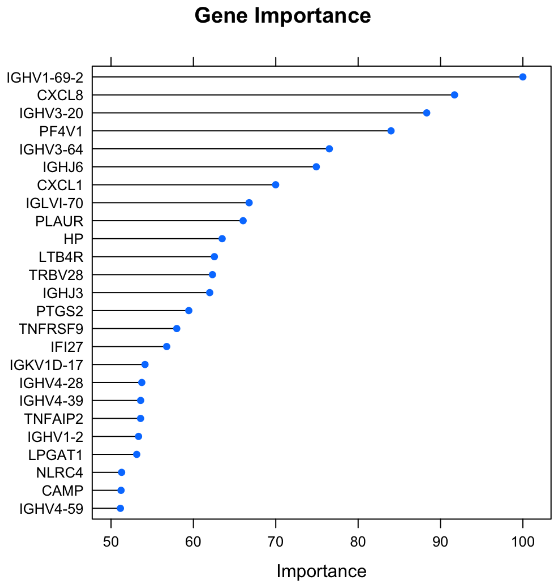

B

Model Type	AUC	Accuracy	Sensitivity	Specificity	Kappa
Logistic Regression	0.93	0.92	0.84	0.93	0.70
GLM	0.97	0.95	0.78	0.98	0.80
SVM	0.90	0.96	0.82	0.98	0.83

Supplemental Figure 7. A machine learning approach predicted AA from a mixture of AA, EA and NAA SLE patients and demonstrated the perturbed B cell axis in AA SLE. (A) SLE patients were classified as African American (AA) using logistic regression, generalized linear models (GLM), and support vector machine (SVM) classifiers. ROC curve for logistic regression and the two different machine learning models in GSE88884 (ILL1 and ILL2 combined). **(B)** Table showing the metrics of three machine learning models. **(C)** Top 25 predictors determined by SVM model.

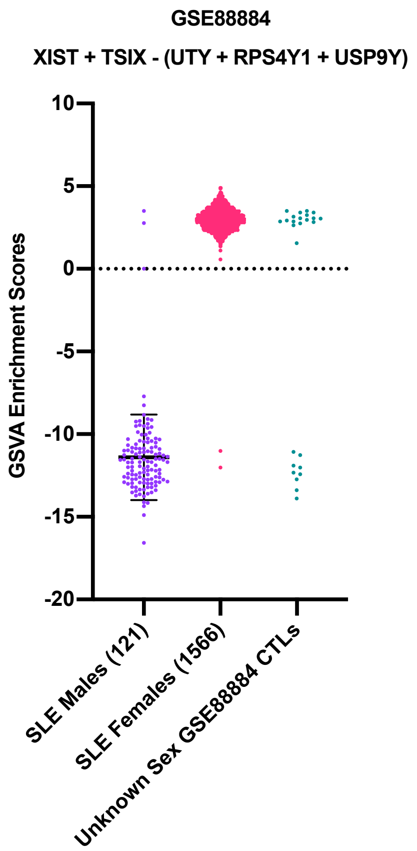

Supplemental Figure 8. Sex module scores for SLE patients listed as males or females and unknown controls in microarray dataset GSE88884. Log2 expression values were used to compute sex module scores using the formula Sex = XIST + TSIX – (UTY + RPS4Y1 + USP9Y). 5 SLE patients (3 male and 2 female) with reported sex in GSE88884 ILL1 dataset were found to have expression of genes consistent with the opposite sex.
